# Complement Activation by Post‐Translationally Modified Proteins: Links to Chronic Inflammation and Autoimmunity

**DOI:** 10.1111/imr.70166

**Published:** 2026-09-16

**Authors:** Marleen M. J. van Greevenbroek, Leendert A. Trouw

**Affiliations:** ^1^ Cardiovascular Research Institute Maastricht CARIM Maastricht University Maastricht the Netherlands; ^2^ Department of Internal Medicine Maastricht University Maastricht the Netherlands; ^3^ Department of Immunology Leiden University Medical Center Leiden the Netherlands

## Abstract

Post‐translational modifications (PTMs) of proteins are essential to maintain homeostasis as many cellular processes rely on reversible PTMs. However, several PTMs, particularly irreversible PTMs in the extracellular space, can contribute to tissue dysfunction, inflammation, and may even trigger the development of autoimmunity against PTM‐proteins. Such disease‐associated PTMs are formed under conditions of metabolic stress, renal failure, smoking, or pollutants. Importantly, several of these PTMs have recently been shown to have the capacity to activate the complement system directly. These PTMs recruit C1q or Ficolin‐3, thereby initiating complement activation. This induces on the one hand opsonins for clearance of (cellular) debris and on the other hand the production of inflammatory mediators. In many autoimmune diseases, patients develop a variety of autoantibodies against PTMs, further amplifying complement activation on PTM‐proteins. Notably, anti‐PTM antibodies are also present, albeit in lower concentrations, in diseases that are not characterized as typical autoimmune diseases. We therefore hypothesize that PTM‐driven complement activation is an underlying factor for chronic low‐level inflammation, thereby predisposing individuals to cardiovascular and metabolic diseases. In addition, the presence of complement activation fragments on PTM‐proteins may facilitate the breakdown of immune tolerance towards PTM‐proteins, further facilitating the production of anti‐PTM autoantibodies. Finally, several interventions are discussed that either decrease the PTM‐proteins or provide targeted complement inhibition specifically on PTM‐proteins. Altogether, complement activation by PTM‐proteins may represent an underestimated contributor to chronic inflammation.

**Abbreviations**
AA: acetaldehyde
AAPA: Anti‐ acetylated protein antibody
ACPA: Anti‐citrullinated protein antibody
AGE: Advanced glycation end products
aHUS: A‐typical hemolytic uremic syndrome
AMD: Age‐related macular degeneration
AMPA: Anti‐modified protein antibody
C3aR: C3a receptor
C4BP: C4b binding protein
C5aR1: C5a receptor 1
CarP: Carbamylated protein
CitP: Citrullinated protein
CVD: Cardiovascular diseases
DAF: Decay accelerating factor
DAMP: Damage‐associated molecular pattern
ER: Endoplasmic reticulum
FHR: Factor H‐related protein
HMGB1: High mobility group box 1
HOMA‐IR: Homeostatic model assessment for insulin resistance
IAPP: Islet amyloid polypeptide
iC3b: Inactivated C3b
MAA: Malondialdehyde acetaldehyde
MAC: Membrane attack complex
MASLD: Metabolic dysfunction‐associated steatotic liver disease
MDA: Malondialdehyde
MGO: Methylglyoxal
NAFLD: Non‐alcoholic fatty liver disease
Ox‐LDL: Oxidized low density lipoprotein
PAMP: Pathogen‐associated molecular pattern
PNH: Paroxysomal nocturnal hemoglobinuria
PTM: Post‐translationally modified
PVAT: Perivascular adipose tissue
RAGE: Receptor for advanced glycation endproducts
SLE: Systemic lupus erythematosus

## 1 Introduction

Following translation from mRNA, proteins can undergo post‐translational modifications (PTMs). Many of these PTMs are part of normal physiological processes while others are associated with pathological conditions and may trigger autoantibody formation. Interestingly, several PTM‐proteins have been shown to directly activate the complement system, a process further amplified in the presence of anti‐PTM antibodies.

In this review we provide an overview of PTMs and discuss the role of PTMs and of anti‐PTM antibodies in disease. We propose a model in which factors present in the intra‐ and extracellular milieu trigger PTM formation, leading to complement activation by the PTM proteins. This, in turn, stimulates inflammation, which triggers the formation of more PTM‐proteins. We compile evidence that PTM‐proteins are an unexpected driver of chronic low‐level complement activation leading to inflammation, one of the key risk factors for the development of cardio‐metabolic diseases and autoimmunity.

## 2 The Complement System

**FIGURE 1:**
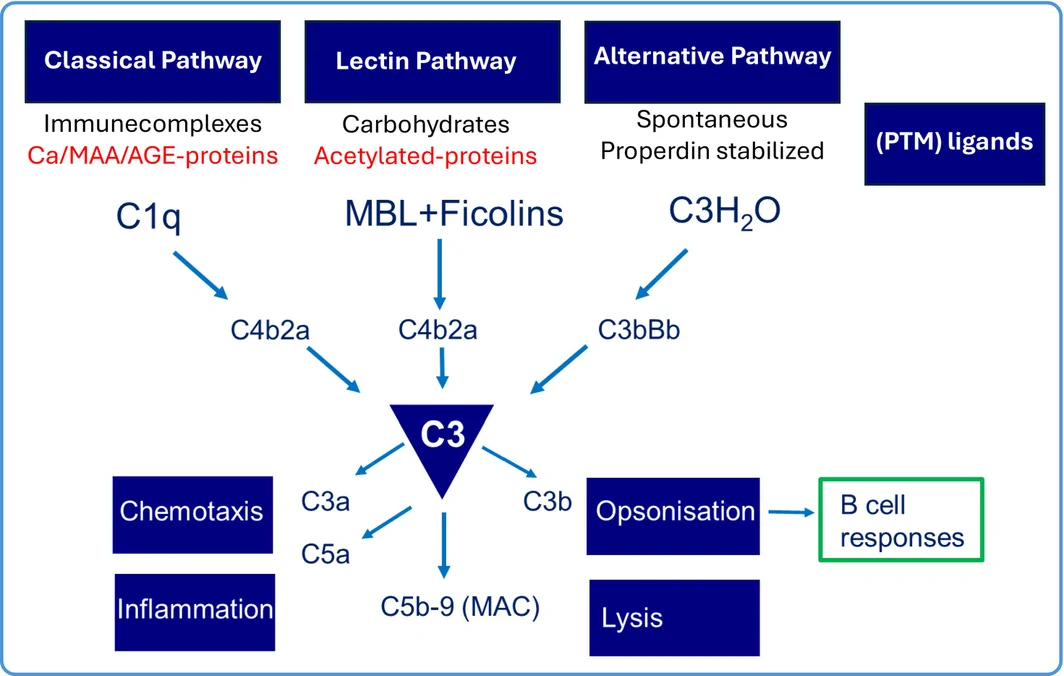
The complement system comprises three activation pathways. Each pathway uses its own recognition molecules that bind to a specific set of ligands, among which are several types of post‐translationally modified proteins (red). Following binding of the recognition molecules to their ligands complement activation proceeds when C3‐convertases are formed that cleave the central component C3 to yield the biologically active fragments C3a, an anaphylatoxin, and C3b that will facilitate opsonization and stimulate antibody responses. If complement activation is sufficiently strong then C5‐convertases will be formed that cleave C5 into the anaphylatoxin C5a, and C5b that will initiate the formation of the membrane attack complex (C5b‐9) that can lyse target cells.

As part of the innate immune defense, the complement system plays an important role in fighting infections [1]. In addition to its antibacterial function, complement also plays a role in instructing the adaptive immune response and in the clearance of immune complexes and dead cells [2]. The complement system comprises nearly 50 proteins, including circulating proteins and enzymes as well as cell‐bound complement regulators and complement receptors [3]. While initially thought to be fully liver derived, it is now clear that next to hepatocytes, many other cell types also produce complement proteins and that some complement proteins are exclusively made by cells other than hepatocytes [3]. The complement system can be activated via three pathways (Figure 1), each initiated by different ligands through different recognition molecules [4]. The classical pathway is activated when its recognition molecule C1q binds to antibody aggregates, to ligand‐bound CRP, or to dead cells. The lectin pathway is activated when its recognition molecules, collectins, including MBL, or ficolins, bind to their ligands such as specific carbohydrates or acetylated proteins. The alternative pathway can be activated spontaneously by a tick‐over mechanism in C3 and is constantly kept in check on human cells by the inhibitory action of complement inhibitor Factor H. Activation of the alternative pathway can be consolidated through properdin and can serve as an amplification loop for the classical and lectin pathways by activating the alternative pathway on C3b generated through other pathways.

Activation of any of these three pathways leads to the formation of C3‐convertases (Figure 1), enzyme complexes that cleave the central component C3 into biologically active fragments C3a and C3b. C3a functions as an anaphylatoxin stimulating inflammation while C3b binds covalently to targets and serves as an opsonin for phagocytosing cells bearing complement receptors. C3b can be degraded to C3d, which has the capacity to bind to Complement Receptor 2 and stimulate B cell responses. When the complement activation is sufficiently strong, the complement cascade proceeds to the formation of C5‐convertases that cleave C5 into C5a and C5b. C5a is another potent anaphylatoxin, further stimulating inflammation. C5b initiates the formation of the membrane attack complex C5b‐9. Insertion of C5b‐9 into the membrane of target cells can cause direct cell lysis. This potentially aggressive system needs to be kept in check in the body to avoid spontaneous, misguided, or overactive activation. Therefore, the body is equipped with a wide array of both fluid‐phase (e.g., C1‐INH, Factor I, Factor H and C4b‐binding Protein) and membrane‐bound (e.g., CD35, CD46, CD55 and CD59) complement inhibitory molecules. The balance between complement activation and complement inhibition is essential to maintain a healthy physiological situation. Excessive complement activation can lead to tissue damage whereas insufficient complement activation will predispose to infections [5]. Such imbalance is seen in several serious human conditions. For example, excessive complement activation by autoantibody‐mediated complement activation triggers organ damage and inflammation in Systemic Lupus Erythematosus (SLE) while mutations in complement inhibitors like factor H resulting in renal complement activation were identified in atypical Hemolytic Uremic Syndrome (aHUS) [1]. In contrast, patients with genetic deficiencies of complement proteins are at high risk to develop a wide array of bacterial infections, a condition also seen in patients under systemic complement inhibitory therapy [1].

## 3 Post‐Translational Protein Modifications and Their Pathophysiological Consequences

**FIGURE 2:**
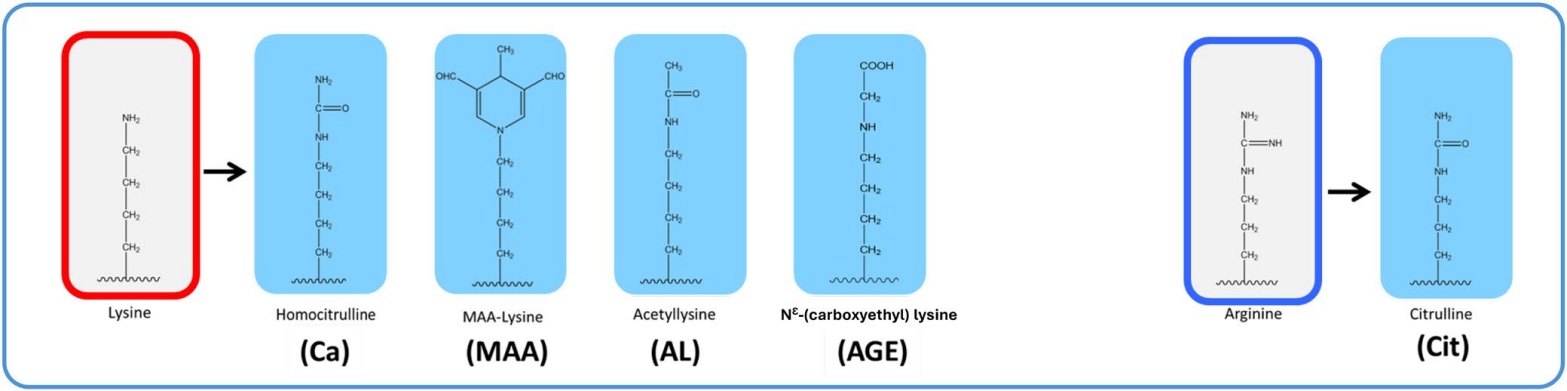
Some amino acids, for example, lysine and arginine, are particularly prone to undergo post‐translational modification (PTM). Multiple PTMs can be formed on the same amino acid, depending on the identity of the modifying agent. Shown in the cartoon are several examples that are discussed in the review. Carbamylation is the process in which cyanate reacts with lysine thereby converting it to homocitrulline. The combined presence of malondialdehyde (MDA) and acetaldehyde (AA) leads to formation of MAA adducts on lysine. Acetylation of lysine generates acetyl lysine. Methylglyoxal (MGO) will generate the carboxyethyl‐lysine (an advanced glycation endproduct, AGE) on lysine residues. Citrullination is the enzymatic conversion of arginine into citrulline. Accessibility of these amino acids for PTM formation depends, among others, on their position in the mature, fully folded proteins.

For maintenance of cellular and systemic homeostasis, proteins in the body are subject to a vast array of post‐translational modifications. These PTMs come in many different forms (Figure 2) but have in common that, after translation of mRNA into mature protein, one or multiple amino acids become biochemically altered.

### 3.1 Post‐Translational Modifications

A wide range of naturally occurring post‐translationally‐induced protein alterations have been identified in physiological and in pathophysiological processes. The post‐translational modifications that are formed on mature proteins can range from enzyme‐mediated and transient to non‐enzyme‐mediated and irreversible. Enzyme‐mediated, transient modifications generally target one specific amino‐acid residue in a specific protein, leading to its activation/deactivation. They include for example, the kinase‐mediated phosphorylation of an enzyme or a receptor and are an indispensable part of normal physiology. An example is the (auto)phosphorylation of components of the insulin signaling cascade [6]. Other types of enzyme‐mediated modifications include methylation, glycosylation, and ubiquitinylation, each with distinct physiological consequences. Together, these enzyme‐mediated, transient modifications comprise > 90% of all reported PTMs [7]. Non‐enzyme‐mediated but reversible modifications are also seen. These include protein S‐sulfenylation, S‐nitrosylation or S‐sulfhydration upon oxidation of Cys, yielding unstable PTMs [7]. These modifications provide a read‐out of oxidative stress and play a role as redox switches [8]. In addition to these reversible protein modifications, there is also a range of broad and irreversible protein modifications. These include, for instance, the advanced glycation end‐products (AGEs) that are formed on multiple amino acid residues in multiple proteins in a state of prolonged hyperglycemia [9, 10]. In general, the formation of specific, transient PTMs is enzyme‐mediated while formation of broad‐spectrum and irreversible PTMs is non‐enzyme mediated. More than 700 different PTMs have currently been identified [7], highlighting the enormous complexity of protein regulation in biological systems.

Non‐enzyme mediated, irreversible PTMs are often, although not exclusively, found in long‐lived proteins. These modifications accumulate over time and are increasingly perceived to be markers of “protein molecular aging” and hence considered hallmarks of aging [6, 11, 12]. Their formation results from the modification of accessible amino acids by reactive metabolites (Figure 2). These reactive metabolites are primarily generated during metabolism and include, for example, methylglyoxal (MGO) produced during glycolysis [12], acetaldehyde (AA) generated in alcohol metabolism [13] and cyanate formed during urea metabolism [14]. In addition, reactive metabolites can arise under conditions of oxidative stress, for example, malondialdehyde (MDA), which is generated during lipid peroxidation of unsaturated lipids in cell membranes [15] (Figure 2). These highly reactive compounds can then modify accessible amino acids, thereby generating PTMs. MGO predominantly modifies mostly arginine (Arg) and lysine (Lys), resulting in the formation of multiple advanced glycation end products (AGEs). MDA and AA together can modify Lys to Malondialdehyde Acetaldehyde (MAA), while carbamylation of Lys by cyanate generates the non‐standard amino acid homocitrulline [15, 16, 17, 18, 19]. While many irreversible PTMs are generated independently of enzymes, one important exception is citrullination, which is the irreversible modification of Arg to citrulline, catalyzed by peptidylarginine deaminase (PAD), particularly in response to high local calcium levels [20, 21].

**FIGURE 3:**
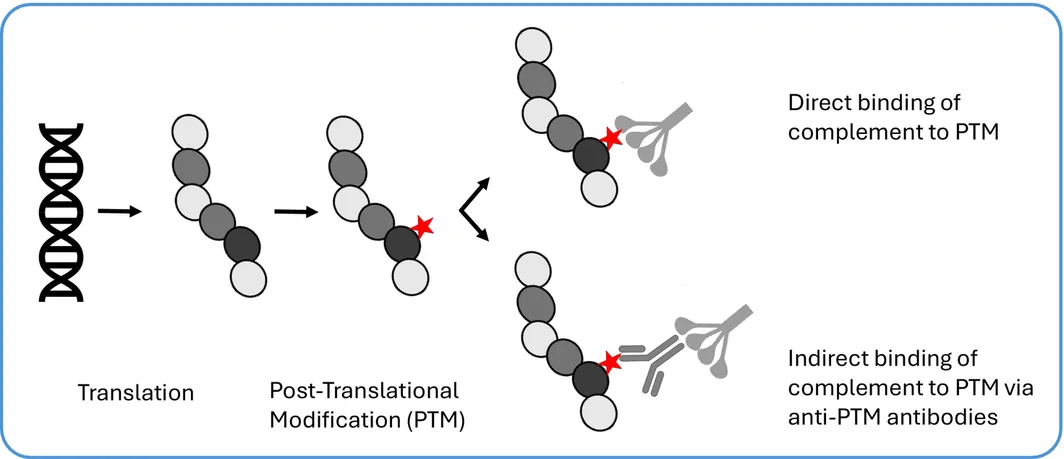
Intracellularly, proteins are generated via transcription of DNA and translation of mRNA. Subsequently, these newly‐formed proteins can undergo a vast range of post‐translational modifications (PTMs). A subset of these modifications occurs in the process of protein maturation, but many additional modifications will take place on the mature protein. These latter modifications can occur intracellularly, but also in the extracellular milieu. Many of the PTMs that are generated on mature proteins are part of normal physiological processes, while others are associated with pathological conditions and may trigger autoantibody formation. Interestingly, several PTM‐proteins have been described that activate the complement system directly, a process further amplified in the presence of anti‐PTM antibodies.

These aforementioned protein adducts or PTMs are generally considered to be chemically irreversible [22]. Removal of PTM proteins can occur via proteasome‐dependent degradation of the proteins whose integrity is affected by the PTMs with subsequent removal of the modified amino acids via the kidney [11, 23, 24]. As discussed below in more detail, circulating PTM‐proteins can also be recognized by complement and/or antibodies that will stimulate clearance via liver and spleen (Figure 3).

The formation of PTMs on proteins can have multiple consequences. Firstly, when the PTM is formed on an amino acid in the functional domain of a protein, it can affect the physiological function of that specific enzyme or regulatory protein. In addition, and likely more relevant for chronic diseases (discussed later), PTMs can interfere with normal physiology through several mechanisms including receptor binding and activation, induction of an antibody response, and via the formation of protein crosslinks. Given that the diversity of PTMs and their (patho)physiological consequences extend far beyond the scope of one review, we herein focus on a subset of irreversible, non‐enzyme mediated PTMs that have been implicated in human diseases that are characterized by a state of chronic low‐grade inflammation.

### 3.2 Post‐Translational Modifications and Disease

Several irreversible, non‐enzyme mediated PTMs including AGE, MAA, carbamylated proteins (CarP), and citrullinated proteins (CitP) have been implicated in a plethora of chronic diseases including, but not limited to, cardiovascular disease (CVD) [25, 26, 27, 28] kidney disease [29], liver disease [30, 31, 32], rheumatoid arthritis [33, 34], and neurodegenerative diseases [20]. Their association with these chronic diseases is not unexpected given the lifelong accumulation of these PTMs. Over the past two to three decades, several mechanisms have been proposed to explain how these PTMs may contribute to chronic low‐grade inflammation and related disease.

Ox‐LDL, a major culprit in the development and progression of atherosclerosis, carries multiple PTMs [35, 36, 37]. Binding of antibodies to ox‐LDL and other PTM tags them for subsequent (complement‐mediated) removal by scavenger cells. While this is basically a beneficial process, it can result in lipid overload of the scavenger cells. In the vascular wall, for example, LDL‐derived lipid accumulation in macrophages results in the formation of foam cells, a hallmark of atherosclerotic plaques.

The intracellular mitochondrially‐expressed C5aR1 has been proposed as a key regulator of the proinflammatory response in human atherosclerotic plaques [38], particularly in male mice. In the context of carbamylation, mass‐spectrometric analyses have been performed to identify the ‘carbamylome’ of the inflamed joint [34]. Carbamylated proteins were readily identified in the synovial fluid, in the synovial tissue and in the cartilaginous tissue. Most of the carbamylation in rheumatoid arthritis was found on the matrix molecules in the cartilage [34], a finding consistent with the concept that the half‐life of matrix molecules is much larger than that of circulating or cell bound protein. This prolonged residence time allows accumulation of PTMs over time.

The protein modifications AGE, MAA, and CarP all bind to the scavenger receptor SR1A on macrophages, eliciting an inflammatory response [39, 40, 41]. The binding of CitP to scavenger receptors and the resulting inflammatory response was enhanced by the simultaneous presence of MAA adducts [42]. AGEs can also activate the Receptor for AGEs (RAGE), which induces oxidative stress and an inflammatory response via, among others, activation of the Nuclear Factor‐kappaB pathway [43]. In addition, PTMs on specific proteins exert pro‐inflammatory effects; for example, the above‐described uptake of PTM‐modified‐LDL via SR‐A1 can contribute to the process of foam cell formation [44], the main initiating step for atherosclerosis. Moreover, citrullinated fibrinogen can activate macrophages via the TLR4 receptor, thereby contributing to an inflammatory response, although this is potentially only as part of an immune complex [45]. Citrullinated proteins have been identified as highly immunogenic, as illustrated by the pronounced presence of anti‐citrullinated protein antibodies (ACPA) in rheumatoid arthritis [46]. Notably, citrullinated proteins are not unique herein, as other PTMs also have been shown to induce an antibody response. Antibodies against AGEs, MAA, and CarP have been identified in patients with auto‐immune disease such as rheumatoid arthritis, but also in patients with CVD [47, 48] and Type 2 diabetes mellitus [49, 50] who were not diagnosed with an auto‐immune disorder.

It remains yet to be clarified to what extent these PTMs and the anti‐PTM antibodies in the circulation are risk factors via separate pathological pathways, whether they are part of a combined pathway, or whether one functions as a biomarker for the other.

### 3.3 Autoantibodies Against Post‐Translationally Modified Proteins

Autoantibodies directed against an endogenous protein are present in many autoimmune diseases. By binding to their target antigen, they can impair protein function, enhance protein function, or cause tissue damage by forming an immune complex and triggering effector functions by cells expressing Fc‐receptors or by activating complement. Over the years it has become clear that some autoantibodies bind specifically to the native protein structure, while other autoantibodies only bind to their target protein after these proteins have undergone a PTM [51].

The term anti‐modified protein antibodies (AMPA) is frequently used to describe the family of autoantibodies that recognize PTM‐proteins [52]. Anti‐PTM antibodies are not only seen in autoimmunity, but also in non‐autoimmune diseases [48, 53]. Several well‐known examples are present in rheumatoid arthritis wherein antibodies were identified that target citrullinated proteins (ACPA) [54]; antibodies that target carbamylated proteins (anti‐CarP) [55], and antibodies against Malonaldehyde Acetaldehyde modified proteins (anti‐MAA) [56]. In other conditions also other PTMs are targeted. For example, autoantibodies against acetylated proteins (AAPA) have been identified, not only in rheumatoid arthritis but also in SLE [57, 58], while antibodies against proteins with AGE‐adducts (anti‐AGEs) were originally described in diabetes [59].

In several diseases, patients can be positive for several anti‐PTM antibodies, hence targeting multiple distinct PTMs simultaneously. This allows clustering of individuals on the basis of their anti‐PTM antibody patterns [57, 58, 60]. The presence of anti‐PTM antibodies is generally associated with worse disease outcomes, for example, more joint damage in rheumatoid arthritis [55, 61, 62]. These anti‐PTM antibodies are reported to have the capacity to contribute to disease severity by triggering cellular activation through Fc‐receptor engagement [63] or by activating complement [64].

Despite our growing knowledge of the widespread presence of anti‐PTM antibodies, it is largely unknown why and how individuals make antibodies against PTM‐proteins and how their relationships are interconnected. Importantly, many of these autoantibodies can already be detected in the circulation of individuals many years prior to the onset of disease [65, 66, 67]. And while there is a degree of cross‐reactivity between anti‐PTM antibodies binding different PTMs [68, 69], clear differences are also observed with individuals being only positive for one anti‐PTM reactivity but negative for others [55, 58, 60]. In experimental animal models, it was shown that immunization with one PTM may trigger antibodies against multiple PTMs [52]. In addition, there may be a certain evolution from one to another anti‐PTM antibody depending on the order of exposures to different PTMs [70, 71].

A key feature that we highlight in this review is the observation that PTM‐proteins can also activate complement [72, 73, 74, 75, 76, 77] (Figure 3 and section 4.1). This provides additional immunostimulatory capacity to the PTM‐protein, thereby enhancing the chance of breaking tolerance. Complement activation not only results in the release of anaphylatoxins C3a and C5a, providing an inflammatory environment resulting in immune cell recruitment and antigen presentation, it also provides C3b/iC3b mediated opsonization. Moreover, C3d, the degradation product of C3b/iC3, can bind to Complement Receptor 2 present on both B cells and on follicular dendritic cells. This is important for antigen presentation and is reported to lower the threshold for breaking tolerance [78].

### 3.4 Technical Pitfalls and Considerations in Identification and Quantification of PTMs and Their Antibodies

The non‐enzymatic PTMs that are the focus of this review are modifications of single amino acids by small reactive metabolites and the resulting changes that are introduced will therefore also generally be small. To identify the non‐enzymatically post‐translationally modified amino acids on proteins, different techniques are available including mass‐spectrometry, ELISA and Western blot. It is important to realize that all these techniques have their benefits and pitfalls when identifying a type of PTM, or the effect of PTMs, on protein function [79]. While not the focus of this review, we highlight here several analytical considerations that may be useful in designing experiments to study PTM‐proteins and their interactions and for the interpretation of data in literature.

First, when producing PTM‐proteins it is important to simultaneously produce the non‐modified version of the same protein using an approach similar to a sham treatment in animal studies. This implies that the unmodified control protein should undergo a mock modification, in which all the reaction steps, the handling, and the incubations are identical *except* for the reagent essential for the modification. This approach ensures that observed effects can be attributed to the PTM itself rather than to differences in protein handling or quality. Second, to allow generalizable conclusions it is important to introduce the PTM to several different proteins because the protein structure and the amino acids surrounding the modified amino acid may influence its binding to anti‐PTM antibodies and/or its interaction with other proteins [77]. Third, when studying complement activation by PTM‐proteins in serum it is essential to also use a control condition consisting of that same human serum but after heat inactivation as a control in parallel experiments. This allows the researcher to evaluate if the presence of complement on PTM‐proteins is the consequence of complement activation or due to passive binding of complement proteins. Fourth, although the identification and quantification by mass‐spectrometry can be very precise, it is important to realize that several PTMs induce a shift in the molecular mass that is actually very similar to mass shifts induced by other modifications. For instance, the identification and quantification of carbamylation is relatively straightforward, whereas the evaluation of citrullination is much more difficult as this very small mass shift closely resembles several other modifications [79]. To be sure that it is actually a citrullinated peptide that is identified by mass‐spectrometry, both modified and unmodified peptides are required to confirm that the spectrum indeed matches the modified peptide. Moreover, some of the PTMs that are stable in their biological, physiological environment are not stable under conditions of sample preparation for mass spectrometry. Fifth, for many of the PTMs commercial antibodies are available, but many of these antibodies cross‐react to other modifications [79]. When ELISA or Western blot are used to identify or quantify PTM proteins, the use of anti‐PTM antibodies to detect the PTM proteins is unavoidable. In this context it is important to realize that the specificity of the detection assay is limited by the specificity of the anti‐PTM antibody that is used for the detection of the PTMs. For example, to enhance the sensitivity of the Western blot‐based identification of citrullinated proteins, the anti‐modified citrulline ‐Senshu method was developed, in which a chemical reaction on the blot combined with specific detection antibodies for this in situ‐modified citrulline enhances the sensitivity of the assay [80]. However, this comes at a cost since this method does not discriminate between citrulline and homocitrulline [81], posing substantial limitations in the interpretation of the data [79]. Sixth, in serum and plasma there is often a simultaneous presence of PTM‐protein and autoantibodies against these PTMs. The presence of autoantibodies against PTMs may interfere with the detection of those PTM, and vice versa, particularly in antibody‐based assays such as ELISA and Western blot. Importantly, the nature and ratio of these circulating PTMs and their antibodies differs according to different disease states. Use of multiple controls and, where possible, confirmation with different detection techniques are advised.

Taken together, these considerations highlight that it is very well possible to quantify PTM‐proteins and to study their interactions with naturally‐occurring autoantibodies and with other proteins in a reliable way, but only with carefully designed experiments and with cautious interpretation of published data.

## 4 Antibody‐Independent Effects of Post‐Translational Modifications on Complement Activation

The above‐described activation of complement via binding of anti‐PTM autoantibodies to their PTM‐protein targets reflects canonical antibody‐mediated activations of the complement system. In addition to this, the formation of PTMs can also affect complement activation via two non‐antibody‐mediated mechanisms. These are (i) the direct recognition of PTM‐protein by complement and subsequent complement activation and (ii) altered functionality of the complement regulators upon the formation of PTMs on complement itself.

### 4.1 Activation of Complement by Post‐Translationally Modified Proteins

Several studies have now described the capacity of PTM‐proteins to activate the complement system directly [72, 73, 74, 75, 76, 77] (Figure 3). Complement activation upon the direct binding of PTM‐proteins differs from complement activation driven by anti‐PTM autoantibodies binding to their PTM‐protein targets [64].

#### 4.1.1 Acetylated Proteins

The first PTM that was identified as a ligand for complement activation was acetylation. Initially, acetylated carbohydrates were identified as ligands for ficolins [73, 74, 76]. Subsequently, acetylated proteins were also identified as ficolin ligands, with preferential binding of purified Ficolin‐3 and considerably weaker binding by purified ficolin‐2 [74]. When serum is used as a source of complement, Ficolin‐3 appears to be responsible for binding and complement activation. The binding of Ficolin‐3 to the acetylated ligand is calcium dependent and results in the activation of the lectin pathway of complement activation. Lindelöf and colleagues described the molecular details of the binding of Ficolin‐3 to acetylated proteins [82]. On suitable targets such as N‐acetylated sugars on pathogens, this will not only generate the opsonins C4b and C3b but will also induce activation of the terminal pathway up to the assembly of C5b‐9. A similar pattern was also reported for acetylated proteins [74]. Another report, focusing on MAA, described that the anaphylatoxin C3a can directly bind to MAA‐modified proteins and thereby contribute to inflammatory processes [83]. Currently there is no clear understanding of how acetylated proteins serve as a target for Ficolin‐3.

#### 4.1.2 Advanced Glycation End‐Products Modified Proteins

The presence of AGEs on glycated proteins was also identified as a PTM that drives complement activation. Using immunoprecipitation and mass‐spectrometric analyses, Chikazawa et al. identified C1q as a specific binding partner to AGE‐modified proteins [72]. C1q was shown to bind to a wide variety of AGE‐modified proteins and to human glycated hemoglobin (HbA1c). C1q binds to the AGE‐modified proteins through the C1q head domains, and the binding is mediated through electrostatic interactions [72]. These observations were confirmed by van den Beukel et al., who showed that complement activation on AGE‐modified proteins is mediated via the classical pathway [77]. Binding of C1q resulted in complement activation, including the deposition of the opsonins, release of anaphylatoxins, and formation of the C5b‐9 complex [77]. It is currently not known which AGE‐modified proteins are the most relevant targets for complement activation, but it is conceivable that circulating AGE‐containing molecules assemble into complexes with complement, as has been shown for immune complexes derived from inflamed kidneys [72]. In addition, cell‐associated and especially matrix molecules may act as more stable long‐term hotspots for ongoing, low‐grade complement activation driven by the AGE‐C1q interaction.

#### 4.1.3 Malonaldehyde‐Acetaldehyde Modified Proteins

The oxidative stress epitope MDA is recognized and bound by a multitude of complement factors including C1q, C3a, Factor H, and factor H‐related protein (FHR) 1, FHR3, FHR5 [84]. The surface‐acting complement activator FHR5, which had been linked to kidney diseases, was shown to bind to MAA epitopes resulting in complement activation. FHR5 activates complement on MAA on the cell surface, thereby competing for MAA epitope binding with the inhibitory complement regulator CFH [85]. In addition, we have shown that higher dicarbonyl stress is associated with a lower plasma concentration of sC5b‐9, and that protein‐bound AGEs were inversely associated with C3a and positively with C5b‐9 in plasma [86], although in these analyses it was not possible to distinguish whether the AGE‐adducts were actually present on complement regulators and on complement targets.

Analogous to systemic complement, intracellular complement—the so‐called “complosome”—can also have protective effects which may relate to regulation of efferocytosis via C3b opsonization in early atherosclerotic lesions [87]. Although not yet specifically investigated, these complosome‐mediated processes may also be relevant in the ectopic fat depots such as the epicardial and perivascular fat depots, similar to what is seen in the atherosclerotic plaque [87].

#### 4.1.4 Carbamylated Proteins

In a comparative study, van den Beukel et al. evaluated the capacity of six different PTMs to activate complement [77]. These include four lysine modifications (carbamylation, acetylation, MAA‐modification and AGE‐modification), as well as one modification on arginine (citrullination) and one on tyrosine (nitration). Complement activation was shown for carbamylation, acetylation, MAA‐modification and AGE‐modification, while no complement activation was detected for nitrated or citrullinated proteins. The data also showed that complement activation on carbamylated, MAA‐modified and AGE‐modified proteins was C1q‐dependent and that complement activation on acetylated protein was mediated by Ficolin‐3. Lindelöf and colleagues highlight that Ficolin‐3 may not only bind to acetylated proteins but may under specific conditions also bind to carbamylated proteins [82].

In the mass‐spectrometric analysis of van den Beukel et al., the complement recognition molecules C1q and Ficolin‐3 were identified as serum proteins that preferentially bound to a PTM‐protein as compared to its non‐PTM control. They also identified downstream complement activation fragments, like C4b and C3b, strongly suggesting PTM‐induced complement activation [77]. Importantly, complement regulators like Factor H were also identified in these mass‐spectrometric analyses in complexes with PTM‐proteins, and presence of factor H on the PTM‐proteins was more pronounced than on the control proteins. In most of these cases, the presence of Factor H depended on previous complement activation, as illustrated by the combined presence of Factor H and C3b deposition on the PTM‐protein. Notably, Factor H binding to the PTM‐proteins was not observed when purified Factor H, rather than Factor H in complete serum, was used. This indicates that on these PTMs the binding of Factor H was indirect, via C3b. There is one important exception: Factor H binds directly to MDA/MAA‐modified proteins [77, 82, 88]. Binding of Factor H to these MDA/AA neoepitopes has a major clinical impact as genetic variants in Factor H that are associated with age‐related macular degeneration (AMD) impair the binding of Factor H to MDA/MAA modified proteins, thereby impacting on overall complement activation and complement‐mediated phagocytosis [89]. The direct binding of Factor H to MDA/MAA modified proteins may serve a role similar to the role of complement activation on apoptotic cells. On these dead cells, some degree of complement activation is beneficial as it will deposit opsonins that enhance clearance, while excessive complement activation, which would lead to C5a release and C5b9‐mediated lysis, needs to be prevented in order to prevent inflammation. This balance between activation and control of complement is mediated by the binding of inhibiting soluble complement regulators such as Factor H to complement‐activation‐complexes [90].

There are small differences in complement levels between individuals of different ages and between the sexes [92]. Irrespective of these variations, a particularly important feature in the context of interaction between complement and PTM‐proteins may be the ‘complotype’ of an individual. The ‘complotype’ refers to the combined presence of a number of single nucleotide polymorphisms (SNPs) in key complement proteins [92]. Each of these SNPs has, by itself, only a small impact on overall complement activity, but their combined presence identifies individuals with a more active or a less active complement system [92]. Interestingly, the presence of these SNPs is also associated with the formation of anti‐PTM antibodies [77], suggesting a relationship between complement activity, binding of PTMs to complement and the formation of anti‐PTM autoantibodies.

Patients with autoimmune diseases frequently have autoantibodies against PTMs but also healthy controls can have some level of autoantibodies. For this reason, it was investigated if PTM‐induced, C1q‐dependent, complement activation was driven directly by binding of C1q or indirectly by PTM autoantibodies. Importantly, purified C1q could also bind to these PTMs, in the complete absence of antibodies. This confirms a direct interaction between C1q and the PTM‐protein [77], as was also shown before for AGE‐modified proteins in an independent study [72]. Complement activation resulted in deposition of the opsonins and continued downstream to the assembly of the C5b‐9 complex.

The observation that not all PTMs activate complement and that the PTMs that do activate complement do not all use the same mechanism support that this interaction is specific. Conceptually, PTM‐proteins have become modified in comparison to their parent protein, which is the original and intended format of that protein. PTM‐proteins deviate from their parents and may therefore be considered inappropriate and targeted for removal. Complement activation with opsonization is a well‐known mechanism to clear unwanted debris, immune complexes, and dead cells, and it fits in this concept that complement activation would facilitate clearance of PTM‐proteins as well. In vitro experiments focused on phagocytosis of PTM‐protein coated beads by macrophages indeed confirmed that the phagocytosis of PTM‐protein coated beads is enhanced by (but not completely dependent on) complement activation on the PTMs [77].

It is striking that not all PTMs on the same amino acid, for example, lysine, have the same complement activation properties. While carbamylation of lysine clearly triggered classical pathway activation by binding C1q, acetylation of lysine triggers lectin pathway activation by binding Ficolin‐3. In addition it is striking that very similar modifications, for example, homocitrulline (the carbamylated lysine) is, as the name implies, very similar to citrulline (the citrullinated arginine) and just one CH2 group longer [51] yet carbamylation does trigger complement activation, while citrullination does not [77].

Epidemiological studies reported an association between presence of AGE‐modified proteins and complement activation [86]. For other PTMs such association studies have not yet been performed. Notwithstanding, for modifications, for example, carbamylation, clear associations have been reported between the levels of carbamylated proteins in the circulation and patient survival in the context of dialysis [93]. Clearly in this complex disease setting of end‐stage chronic kidney disease (CKD) several (immunological) pathological processes [93] may be operational at the same time, but chronic low level complement activation triggered by the presence of carbamylated proteins may contribute to the poor outcome in the subset of diseased individuals with high levels of carbamylation. It is well known that complement is activated in patients with end‐stage kidney disease either on hemodialysis or peritoneal dialysis [94]. It is conceivable that PTM‐proteins, accumulating in these individuals as a consequence of inflammation and increased urea levels, contribute to complement activation and to chronicity of the low‐grade inflammation.

The nature and physical location of PTM‐proteins will have a major impact on how these PTM‐proteins can activate complement and how that will influence the inflammatory response. If PTMs occur on circulating proteins, for example, albumin, then it is likely that there need to be multiple modifications present on one molecule to trigger complement activation, as for example, C1q will have to bind with several of its ‘arms’ to ligands to become activated. If complement activation takes place, then opsonization will enhance their clearance. A very different scenario is the modification of matrix molecules where the modifications can more easily occur in close proximity to each‐other, thereby facilitating complement binding. The same will hold true for complement binding to protein deposits such as amyloid in Alzheimer's dementia. In these settings, opsonization cannot lead to clearance and an ongoing process of chronic complement activation and inflammation will take place. Inflammation is a powerful driver of additional PTM formation (e.g., MAA, acetylation and carbamylation) as well as the production of complement proteins, resulting in a self‐perpetuating circle of complement‐mediated inflammation.

For patients suffering from autoimmune diseases, it is well known that they may be positive for one or even several anti‐PTM antibodies to distinct PTMs [57, 58, 60]. In this setting the complement activation is more complex as both the PTM‐protein and the anti‐PTM antibodies can trigger complement activation (Figure 3). It is well known that several anti‐PTM antibodies can activate complement; for example, ACPA [64, 95], anti‐CarP [96], anti‐MAA [97] and anti‐acetylated protein antibodies [75] have been reported to do so. While for other anti‐PTM antibodies no formal reports describe their complement activating potential, it is conceivable, given the isotype use, IgM and IgG1 and IgG3, that most anti‐PTM antibody responses will have this capacity. There are, however, several additional factors that influence the degree of complement activation by anti‐PTM antibodies, as both the avidity of the anti‐PTM antibodies [98] and the presence of Fab‐glycans [95] can impact on the capacity to activate complement.

Our unpublished work indicates that the degree of complement activation triggered by the anti‐PTM antibodies is larger than that triggered by PTM‐proteins themselves. However, formal comparative studies will be required to support that notion for a variety of modifications.

### 4.2 Post‐Translational Modifications on Complement Proteins

Besides activation of complement via the above‐mentioned binding of complement recognition molecules to PTM‐proteins, PTMs can also be formed on the complement factors and complement regulators themselves. Human serum complement components, including C3, can be carbamylated in vitro [99], indicating that in their native conformation at least part of their lysine residues are accessible for post‐translational modification. Note that the overrepresentation of information on C3 in this literature on PTMs on complement is likely due to its relatively high concentration in plasma which facilitates the detection and analysis of PTMs that are formed on this molecule. Another example is glycation (i.e., the formation of AGE‐adducts) on CD59, which is the complement inhibitor that prevents C5b‐9 assembly. Glycation of CD59 has been shown to impair its protective function, as reflected in increased deposition of C5b‐9 in the kidneys in diabetes patients [100]. Similarly, glycation also alters the activity of decay accelerating factor (DAF/CD55) in persons with diabetes [101]. Moreover, hyperglycemia‐induced AGE‐adducts may play a role in the C3‐mediated resistance to fibrinolysis of blood clots in diabetes patients [102, 103].

Taken together, these data show that the formation of PTMs on complement proteins, particularly when they are generated in the active sites of complement regulators, can disturb the delicate balance between complement activation and inhibition.

## 5 Impact of Complement Activation on Risk for Disease and on Disease Progression

The activation of complement, via either of its three major activation pathways (the classical, the lectin, and the alternative pathways), will generally result in an inflammatory response mediated via the anaphylatoxins (C3a and C5a), the opsonins (C4b and C3d), and the effector molecule of the terminal pathway (sC5b‐9). Besides via the three major pathways, complement can also be activated via interplay with the extrinsic proteases of the coagulation, fibrinogen, and kinin systems. Activation of complement components via these non‐canonical pathways also contributes to the role of complement in disease. Local as well as systemic complement activation plays a role in many non‐communicable diseases. Complement perturbations contribute to a wide range of acute and chronic diseases related to low‐grade inflammation. These include, but are not limited to, obesity‐related cardiometabolic diseases such as CVD and Type 2 diabetes mellitus, autoimmune diseases such as rheumatoid arthritis and neurodegenerative diseases such as cognitive decline and dementia, as well as their vascular comorbidities.

### 5.1 Complement in Adipose Tissue

Adipose tissue is nowadays accepted as a key regulator of metabolism and homeostasis. Oxidative stress, particularly in the visceral and other ectopic depots, is a hallmark of dysregulated adipose tissue metabolism [104]. Nutrient excess may overcharge the intrinsic metabolic capacity of adipocytes thereby inducing endoplasmic reticulum (ER) stress, mitochondrial dysfunction, and oxidative stress [105]. The oxidative stress will be further aggravated through the presence of the damaged mitochondria [106]. Moreover, hypoxia that occurs in the enlarged adipose tissue depots in obesity may confer intracellular stress, thereby eliciting an inflammatory response [107]. The adipose tissue produces an array of complement components, particularly components of the classical and the alternative activation pathways. Obese individuals have increased circulating concentrations of C3a and C5a which elicit an inflammatory response [108]. Complement activation in dysfunctional adipose tissue links obesity‐induced systemic derangements including low‐grade inflammation, insulin resistance, and dyslipidemia [109]. The liver can also contribute to the obesity‐induced increase in circulating complement. The liver is a major source of systemically present complement factors, including the central component C3. The liver is also directly exposed to exogenous (dietary) fatty acids from the intestine and endogenous fatty acids from the visceral adipose tissue which can overload the hepatocytes leading to hepatic fat accumulation and inflammation. Complement production in the liver is increased in conditions where fat accumulates in the liver such as metabolic‐associated fatty liver disease (MAFLD) [110], previously referred to as non‐alcoholic (NA) FLD [111].

Control and function of the complement system depend on a delicate balance [5] which, once disturbed, will perpetuate to affect different cardiometabolic systems. There are several reasons why the intrinsic balance of the complement system may be particularly vulnerable to obesity‐induced metabolic changes. First, many complement components are synthesized in adipocytes and in the stromal cells of the adipose tissue; second, their expression and production are altered in obese adipose tissue; third, also plasma concentrations of multiple complement components are increased in obesity, although it is not known if this is via direct production and secretion of complement by the adipose tissue, or via indirect pathways (e.g., the liver). Notably, adipose tissue, particularly in obese individuals, is not a single depot but is partly deposited as ectopic depots adjacent to many organs including, but not limited to, perivascular, epicardial, perirenal as well as intrahepatic and intramuscular fat depots [112]. Concentrations of certain complement factors are associated with fat accumulation in general, while others are more strongly associated with these visceral and ectopic fat depots [109]. Dysfunctional adipose tissue metabolism can also affect body composition. In rheumatoid arthritis this is reflected in a relative increase in ectopic fat combined with a decrease in muscle mass, reminiscent of what is seen in aging [113]. Pericardial fat in rheumatoid arthritis is associated with cardiovascular disease [114]. Moreover, in experimental mouse models, C3 and C4 contributed to aorta stiffness through thiol‐ether binding to collagen and elastin in the vascular wall [115]. These authors pointed at the perivascular adipose tissue (PVAT) depot that surrounds the aorta as the most likely source of these complement components and suggested that PVAT‐derived complement may contribute to “outward‐in” remodeling of the vasculature. Likewise, PVAT‐derived C3 was shown to modulate the migration and differentiation of adventitial fibroblasts [116]. Complement has also been implicated in remodeling of the non‐vascular extracellular matrix in a range of other organs including the eye [117], the lung [118] and the kidney [119].

### 5.2 Complement and the Inflammasome

Chronic low‐grade inflammation lies at the core of multiple diseases including cardiometabolic, neurodegenerative and autoimmune disease and their comorbidities. Activation of the inflammasome is a major pathway via which complement activation contributes to inflammation. Complement and the inflammasome are the two major danger‐sensing effector systems of innate immunity. An extensive overview of their key roles in the inflammatory response was recently published [120]. Via bidirectional crosstalk, complement and inflammasome activation are mutual amplifiers: An activated complement can sensitize and induce inflammasome activation while the effector molecules of the inflammasome, for example, IL‐1β, induce the production of complement [120]. C3a and C5a [121, 122] and sublytic C5b‐9 [123] can all activate the NLRP3 inflammasome in macrophages thereby contributing to an inflammatory response and ROS production. Complement activation has been implicated in the initiation of inflammation via activation (e.g., via binding of C3a and C5a to their receptors) and inhibition (e.g., via CD46) of the NLRP3 inflammasome [121, 122, 124, 125]. Sublytic C5b‐9/MAC on immune cells is another complement‐derived inflammasome‐activating signal [123, 126]. The classical pathway initiator C1q has been implicated in promoting as well as mitigating inflammasome activity, depending on the context. C1q was reported to contribute to inflammasome activation in disease states such as rheumatoid arthritis [127], Alzheimer's dementia [128] and stroke [129, 130]. On the other hand it prevents inflammasome activation through its role in the clearance of apoptotic cells and the concomitant induction of an anti‐inflammatory response in macrophages [131], as was seen in the early stages of atherosclerosis [132]. In obesity and MAFLD, small amounts of gut‐derived lipopolysaccharide (LPS) can sustain a state of low‐grade inflammation [133], at least in part via non‐canonical inflammasome activation, with direct activation of caspase [134]. Microcrystals and extracellular ATP can activate both complement and the inflammasome [135, 136]. Activation of the recently identified complosome, the intracellular complement system, can also contribute to inflammasome response via directly activating the inflammasome and via lowering its activation threshold [136, 137]. The joint stimuli for activation of complement and the inflammasome include danger‐associated molecular patterns (DAMPS) and pathogen‐associated molecular patterns (PAMPS), mitochondrial damage, cellular stress and particle matter [120]. Notably, these DAMPS will include PTM. Cell stress can be a consequence of PTMs on cellular protein and PTMs can also induce the accumulation of particulate matter such as amyloid Aβ in Alzheimer's dementia. The receptor for AGE (RAGE) is a key pattern recognition receptor that induces the inflammasome [138]. Note that the cycle of amplification between complement and the inflammasome will be more prominent in the context of a relative shortage of functional complement inhibitors.

### 5.3 Complement in Chronic Low‐Grade Inflammation: Focus on Cardiometabolic Disease, Neurodegeneration and Rheumatoid Arthritis

Given their close co‐existence and the intricate relationship between complement activation and inflammation, it is hard to determine the exact order of events for their effects on the development of low‐grade inflammation‐related diseases. We and others have shown that circulating complement activation products such as C3a, C5a, and soluble C5b‐9 are associated with systemic low‐grade inflammation [109]. Early experimental studies in mouse strains that were genetically deficient for the receptors C3a and C5a support that complement activation is essential for, and precedes, the development of high fat diet‐induced adipose tissue inflammation and insulin resistance [139, 140]. An early study by Muscari et al. identified systemic C3 as a risk factor for incident CVD [141]. In line with this, higher plasma concentrations of C3 predicted future weight gain in a large observational human study that included only male participants [142], and we showed that C3 (and C4) concentrations were associated with incident metabolic syndrome [143]. C3 is also associated with the development of incident Type 2 diabetes mellitus, independent of the degree of low‐grade inflammation [144]. At present, possibilities for pharmacological modulation of complement activation on diseases related to low‐grade inflammation are limited given the high cost of the currently available compounds and high chance of unwanted side effects due to systemic chronic complement inhibition.

C1q and C1q‐induced phagocytosis are considered anti‐inflammatory, which is illustrated by the observation that C1q limits inflammasome activation in macrophages [131]. These effects of C1q are consistent with the finding that C1q deficiency is associated with autoimmunity [145]. However, while generally considered to induce an anti‐inflammatory state, C1q can also provoke a pro‐inflammatory response when activating the downstream effectors of the complement cascade including the amplification loop and the terminal pathway. C1q was shown to contribute to ongoing local inflammation in Alzheimer's dementia and atherosclerosis, among others by binding to oxidized (i.e., post‐translationally modified) LDL and steering the attraction of immune cells [146]. This classical pathway‐mediated proinflammatory process was ameliorated by the binding of lipoprotein apoE to C1q [146]. C1q can also, via its globular head domains, bind to the receptor for AGEs (RAGE) thereby enhancing C1q‐mediated phagocytosis [147], reflecting an effect of C1q that is independent of the activation of complement. RAGE is a multiligand receptor and activation of RAGE on adipocytes and macrophages which, via its canonical ligand, AGE, induces a proinflammatory response [9]. Binding of another RAGE‐ligand, that is, the high mobility group box 1 protein (HMGB1) which is an intracellular danger system released by apoptotic cells to RAGE on macrophages, also induces a proinflammatory response and an M1‐like macrophage phenotype [148]. Notably, this response is modulated by C1q wherein the combined binding of C1q and HMGB1 to RAGE on monocytes induces an anti‐inflammatory M2‐like phenotype [149]. More specifically, binding of HMGB1‐only, in the absence of C1q, results in RAGE‐mediated leukotriene production, while binding of HMGB1 plus C1q induces the production of the specialized pro‐resolving lipid mediators lipoxin A4, resolvin D1, and resolvin D2 through a RAGE‐ and LAIR‐1‐dependent pathway [150]. It remains to be evaluated if C1q can also modulate the effect of AGE‐induced RAGE signaling.

These dual and opposing effects of C1q on phagocytosis and inflammation may underlie the U‐shaped relationship of systemic C1q levels with incident CVD that we observed in a human cohort [151]. Notably, a similar U‐shaped relationship was reported for the association of MBL with carotid intima‐media thickness in patients with rheumatoid arthritis [152]. It appears that—at least locally, at the tissue level—there is an optimum for complement activation, balancing the protective effects related to the removal of dead cells, debris, and protein depositions with the detrimental effects that likely result from ‘too pronounced’ local complement activation and the subsequent inflammatory response. Thus far, such U‐shaped relationships were not reported for the activation of the alternative pathway in human studies. In fact, activation of the alternative pathway was generally associated with the development of disease. Factor D, the rate limiting enzyme in the activation of the alternative pathway, was in a large prospective cohort (men only) shown to be associated with stroke [153] but not with coronary heart disease [154]. In a cross‐sectional analysis of an unrelated cohort, we showed that factor D is positively associated with low‐grade inflammation and endothelial dysfunction which translated to more CVD, manifesting primarily as cerebral vascular disease in the male participants [155]. This difference between the initiators of the classical and the lectin pathway versus the activation of the alternative pathway makes sense given the prominent roles of the former in direct opsonization and phagocytosis and the generation of the prominent anaphylatoxin C3a in the latter.

In experimental models, antagonists of C3aR and the C5aR1 attenuate development of dementia and atherosclerosis in suitable models [156, 157]. Some complement‐mediated effects on disease may be sex‐specific, as illustrated by the results of an experimental mouse model showing that deletion of C5aR1 results in a reduction of atherosclerosis in male, but not in female mice [158]. The cholesterol crystals in the atherosclerotic plaques are coated with complement, suggesting that they are primed for complement‐mediated phagocytosis while concomitant generation of anaphylatoxins triggers the (local) inflammatory response [122, 159]. The observation that activation of C3, C5, and the terminal pathway was – in experimental mouse models‐ consistently shown to be proatherogenic suggests that the observed protective effects of the classical and the lectin pathway in atherosclerotic disease lie in their proximal steps.

An emerging comorbidity of (obesity‐associated) cardiometabolic diseases such as CVD and Type 2 diabetes is neurodegeneration, reflected in cognitive decline and dementia, which may at least partly be attributable to systemic and local inflammation, including neuroinflammation, but also to underlying vascular pathology, as in vascular dementia. Complement may contribute to these pathological processes via local production and activation of complement and via induction of endothelial injury [160]. Local complement activation was reported in the key lesions involved in the pathology of these diseases, that is, atherosclerotic plaques in the vascular wall [161], amyloid Aβ plaques in the brain [162] and islet amyloid polypeptide (IAPP) in the pancreatic beta cells [163]. Accumulation of intra‐ an extracellular debris can act as a damage signal which is detected by complement and subsequently triggers an enhanced inflammatory signal. Experimental mouse models suggested a role for the C3‐C3a‐C3aR axis in Aβ‐induced dementia [164], as well as in disruption of blood–brain barrier integrity [165]. Moreover, concentrations of C3 in plasma and cerebrospinal fluid are higher in patients with Alzheimer's Dementia than in controls [166] and plasma C3 predicts CVD in Alzheimer's dementia [167]. In contrast, low as opposed to high C3 concentrations also associated with a higher risk to develop Alzheimer's dementia, especially in carriers of the E4 allele [168]. Likewise, there is experimental data suggesting the complete absence of full complement activation through C3 deficiency is detrimental as well, because C3 deficiency resulted in accelerated disease in a dementia‐prone [169] and in an atherosclerosis‐prone [170] mouse model. These latter effects might be related to the proposed role of complement in neuroplasticity and repair in normal neuronal development [171].

Vascular calcification is yet another disease state that is characterized by accumulation of insoluble deposits. Prevalence of vascular calcification increases with age, is particularly high in chronic kidney disease [172] and is also a common comorbidity in rheumatoid arthritis [173]. In the process of vascular calcification, calcium is deposited in the vascular wall, thereby contributing to the stiffening of the arteries. Vascular stiffness and vascular calcification are multifactorial. An array of complement factors, indicative of overall complement involvement and activation, was identified in the calcified microdomains in the affected arteries [174, 175], and the plasma concentration of C3a was also associated with abdominal and coronary calcification [176, 177]. It is not yet clear if, as has been suggested, complement actively and directly contributes to the calcification process via osteogenic transdifferentiation of vascular smooth muscle cells [178] or rather that complement is activated on the surface of the hydroxyapatite crystals, similar to what is seen for cholesterol crystals in the atherosclerotic plaque [122].

### 5.4 Complement in Healthy and Unhealthy Aging

With increasing age, the balance in complement activation shifts towards a state of less rigorous checkpoint control by complement regulators and consequently towards a state of continuous ‘low‐grade’ complement activation that contributes to the process of ‘inflammaging’ [171]. In the process of healthy aging, such a state of mildly enhanced complement activation may boost the immune surveillance role of the complement system in defense and repair, which might be beneficial in the prevention of aging‐related diseases [179]. Notwithstanding, complement activation has extensively been implicated in progression of diseases of the elderly. C1q increases strongly with increasing age and complement activation via the classical pathway has been suggested to contribute to sarcopenia [180], a condition that is also common in rheumatoid arthritis [181]. In experimental models it was shown that during aging, chronic complement activation and signaling via C3aR contributes to deterioration of the integrity of the blood–brain barrier [165] and signaling via C5aR1 contributes to neuroinflammation and progression of cognitive decline [156]. At the same time, complete absence of C3 in animal models of Alzheimer's dementia also associated with worse neurodegeneration and more Aβ amyloid accumulation, suggesting compromised clearance of amyloid deposits [169].

Taken together, there are some positive effects of complement and complement activation that, when preserved, will protect from development of disease during healthy aging. On the other hand, tipping of this balance towards the non‐beneficial effects of advanced complement activation likely results in a state of ‘unhealthy and accelerated aging’ and to the development of (aging‐related) chronic diseases.

## 6 Intervention in Post‐Translational Modification‐Driven Disease

The presence of PTM‐proteins is associated with a higher risk to develop cardiometabolic and autoimmune diseases and several lines of experimental evidence support a connection between the presence of PTM‐proteins and chronic inflammation. As a result, there is growing interest in developing therapeutic strategies to intervene in these processes. Such approaches may target prevention of PTM formation or target clearance of PTMs, once formed. Potential strategies can include modification of lifestyle and/or environmental exposure, which can be either broad or focused. Alternatively, therapeutic strategies may target the modulation of the complement activation that is driven by PTM‐proteins and/or anti‐PTM antibodies. In this latter approach, using complement inhibitors directed to the PTMs represents a potential avenue to reduce inflammation while preserving essential immune functions.

### 6.1 Intervention Through Lifestyle and the Environment

Accumulation of PTMs during the course of life is, among others, driven by a person's life‐long environmental, lifestyle and biological exposome. The environment contributes to PTM formation for example, through air pollution [182], while lifestyle components such as exercise, diet and alcohol intake, or smoking habits also have profound effects on PTM formation. They thereby contribute to the biological exposome which is further shaped by genetic and epigenetic factors.

Oxidative stress is a common consequence of an unfavorable environmental, lifestyle and biological exposome. It is characterized by the presence of reactive oxygen species (ROS) which induce the generation of reactive carbonyl species, one of which is MDA [15]. Moreover, intracellular metabolism of glucose (glycolysis) generates MGO as a by‐product [12]. MDA and MGO are highly‐reactive metabolites that will readily engage in the generation of MAA and AGE, respectively. This suggests that reducing the bioavailability of these highly‐reactive catalysts of PTM formation is an attractive target to control the production of PTMs. One potential approach to reduce the bioavailability of MDA and MGO is supplementation with dicarbonyl scavengers such as the vitamin B6 vitamer pyridoxamine [12, 183] or the flavonoid polyphenol and antioxidant quercetin [184, 185]. Additional, less focused approaches are regular moderate exercise and a healthy diet such as the Mediterranean diet, both of which may help reduce the total oxidative stress burden [25, 186, 187]. Moderate‐to‐high alcohol consumption can also add to oxidative stress and detoxification of alcohol in the liver generates AA, which combines with MDA to generate MAA. This makes reduction of alcohol consumption an attractive potential strategy to reduce MAA‐adducts. Moreover, cigarette smoke has been identified as a direct source of AA [188], which makes smoking cessation an opportunity to reduce MAA formation in smokers. Also for carbamylation, smoking is reported as an important contributor, so smoking cessation will be beneficial to reduce the exposure to many PTMs [19].

The dietary measures that are available to reduce urea in the circulation to prevent the formation of carbamylated proteins basically boil down to the general recommendations for management of kidney function including optimal hydration, moderate intake of protein and salt, and high fiber intake. AGEs are present in foods, particularly in (highly) processed foods [189]. To prevent the accumulation of AGEs, an additional strategy lies in the prevention of their entrance into the body. Herein, it must be noted that it is not yet known if the biological effects of exogenous (i.e., ingested via the diet) and endogenous (i.e., generated inside the body) AGEs are comparable. Notwithstanding, a recent meta‐analysis of 14 human interventions with diets high and low in AGEs showed, despite high heterogeneity, an overall detrimental effect of dietary AGEs on metabolic qualifiers of Type 2 diabetes mellitus that is, fasting glucose, fasting insulin, HOMA‐IR [190]. Notably and perhaps counterintuitively, recent observational studies on the effect of MGO in the diet showed a beneficial effect of higher MGO intake on incident Type 2 diabetes mellitus [191] and CVD [192]. The mechanism for this has not yet been elucidated but might be related to hitherto unanticipated beneficial effects of MGO on the gut microbiome.

Taken together it appears that for mitigating the accumulation of non‐enzymatic irreversible PTMs, following the general advice for a healthy lifestyle as recommended by the WHO [193] is a sensible approach. Whether the addition of specific compounds such as scavengers of PTM precursors will be of added value, particularly in persons at a higher risk of developing chronic non‐communicable diseases, needs further evaluation in future randomized controlled trials.

### 6.2 Medical Interventions—Towards Therapeutic Antibodies

The treatment of autoimmune diseases, including those characterized by the presence of AMPA, is largely based on non‐specific systemic immunosuppression. This type of treatment does alleviate the symptoms for the patients, reduces inflammation and limits organ damage, yet rarely leads to cure, and importantly for this review, it does not lead to a reduction in the disease‐provoking AMPA [194]. Even B cell‐targeted therapies, although effective in for example rheumatoid arthritis, do not lead to disappearance of AMPA [195]. Other treatments, such as plasmapheresis, may give a temporal drop in AMPA levels but do not address their production and hence AMPA will likely return after treatment. Novel therapies, derived from the oncology field, use Chimeric Antigen Receptor T cells (CAR‐T cells) to kill all the B cells in a patient [196]. This treatment was successfully tested in patients suffering from severe treatment refractory SLE and is now tested also in many other rheumatic conditions. Following systemic B cell depletion in these SLE patients, there was a complete disappearance of autoantibodies that also did not reappear once new B cells were generated [196]. Also, the symptoms of these individuals disappeared, indicating a state of cure in severe refractory SLE [196]. Because this treatment eliminated all autoantibodies in these SLE patients, it is likely that also AMPA were reduced, but so far no papers specifically reported on this. At present, only a few reports on treatment with CAR‐T cells of rheumatoid arthritis patients who were unresponsive to conventional treatment showed clear disappearance of ACPA [197, 198]. For other AMPA, this has not yet been studied, but it is conceivable that all AMPA will respond similarly to this CAR‐T treatment. Clearly, broad application of CAR‐T cell therapy is currently still limited because of the invasiveness of this intervention, with serious risks, side effects and major costs.

There are currently around 14 inhibitors on the market that are directed against 8 different complement targets [199]. These drugs are mainly used in rare diseases that are not antibody‐mediated, such as atypical Hemolytic Uremic Syndrome (aHUS) and Paroxysmal Nocturnal Hematuria (PNH). Only two of the currently approved complement inhibitory drugs are used in autoantibody driven diseases like Cold Agglutinin Disease and ANCA vasculitis [1]. However, these diseases are driven by antibodies against erythrocytes and neutrophil proteins, respectively, and are not diseases typically driven by PTMs or by anti‐PTM antibodies. Systemic complement inhibition is currently also tested in several trials in SLE, and some small trials have been performed in rheumatoid arthritis, but so far with disappointing results, in combination with unfavorable increase in the risk of infections [200].

To enhance complement inhibition while mitigating the unwanted increase in infections, several strategies are being developed to move away from systemically acting complement inhibitors towards targeted local complement inhibition [201, 202]. These approaches can roughly be divided into 2 lines of complement inhibitors. Firstly, an approach in which the complement inhibitors localize to tissue‐bound complement activation fragments, so the place where complement has already been activated, where they can inhibit further downstream activation [201, 203]. And secondly an approach in which complement inhibitors are specifically targeted to specific tissues or antigens where they can inhibit ongoing complement activation and prevent complement activation from occurring [202]. The latter approach has now been used to bring complement inhibitors specifically to PTM‐proteins. One line of research is focused on targeting complement inhibitors specifically to ischemia‐related neo‐antigens, as reviewed by Tomlinson [204]. The other line of research is focused on the use of bi‐specific antibodies to bring endogenous complement inhibitory proteins to specific PTM‐modified tissues or PTM‐proteins [96, 202, 205]. In these bispecific antibodies one arm of the antibody binds to a disease‐related tissue antigen while the other recruits one of the circulating endogenous complement inhibitors, Factor H or C4b‐binding protein (C4BP) [202]. As a proof‐of‐concept, the first bi‐specific antibodies were directed against model antigens such as dinitrophenyl (DNP) and biotin, but recently the first PTM‐reactive bi‐specific antibody has been made based on an anti‐CarP antibody, targeting carbamylated proteins [96]. These in vitro data indicate that it is indeed possible to bring the complement inhibitors Factor H or C4BP in close proximity to carbamylated proteins and in addition to inhibit local complement activation that was induced by anti‐CarP autoantibodies derived from rheumatoid arthritis patients [96]. These approaches to achieve local targeted complement inhibition have the potential to allow effective inhibition at the site where pathological complement activation is taking place while leaving the systemic complement system intact to fight infections elsewhere in the body [202]. This results in a much more favorable risk profile, and likely reduced costs, thus allowing complement inhibitory treatment in a wider set of clinical conditions. These bi‐specific antibodies may also allow for interventions in conditions where PTM‐driven complement activation contributes to chronic low‐level inflammation, for example, by targeting the endothelium. The modular nature of bispecific antibodies allows exchange of the anti‐CarP targeting arm for another anti‐PTM targeting arm. In addition, because of the cross‐reactive nature of some anti‐PTM antibodies, it may be possible to design a bi‐specific antibody that will target more than one PTM.

Collectively, changing lifestyle and environmental factors will reduce the generation of new PTM‐proteins and this reduction may be further enhanced by supplementation with several types of scavengers. In addition, targeted therapeutic interventions may reduce local complement‐driven inflammation that is induced by PTMs and dampen the vicious circle of chronic low‐grade inflammation as a driver of cardiometabolic and autoimmune disease and the generation of additional PTMs.

## 7 Knowledge Gaps

There is substantial biochemical evidence demonstrating that proteins that carry PTMs can directly activate complement. In several diseases, anti‐PTM autoantibodies are present, and these antibodies further enhance complement activation. Epidemiological data support a link between metabolic stress and the formation of PTMs, complement activation, and inflammation. Together, these different observations support a relationship in which the formation of PTM‐proteins can trigger complement activation, thereby contributing to chronic low‐level inflammation, a key risk factor for cardiometabolic and autoimmune disease. In addition, the complement activation by PTM proteins may facilitate a break in tolerance and the production of anti‐PTM antibodies in a subset of genetically predisposed individuals, which represents a further risk factor for cardiometabolic and autoimmune disease. Inflammation is a key driver of PTM formation, facilitating the vicious circle of PTM formation, complement activation, inflammation, and continued PTM formation. However, not all observations are currently connected through experimental and/or epidemiological evidence, and many points are still unknown, including—but not limited to: What are the disease‐relevant PTMs? Which modified proteins play key roles in pathogenesis? What environmental factors drive disease‐relevant PTM formation? What interventions can reverse this process? What is the relative contribution of PTM‐driven complement activation in chronic inflammation? Can we inhibit this process in vivo?

By systemically answering these open questions we will gain a much deeper understanding of the risk factors that contribute to the onset of several major human cardiometabolic and autoimmune diseases and reveal the possibilities to intervene in a preventive and therapeutic setting.

## 8 Concluding Remarks and Outlook

**FIGURE 4:**
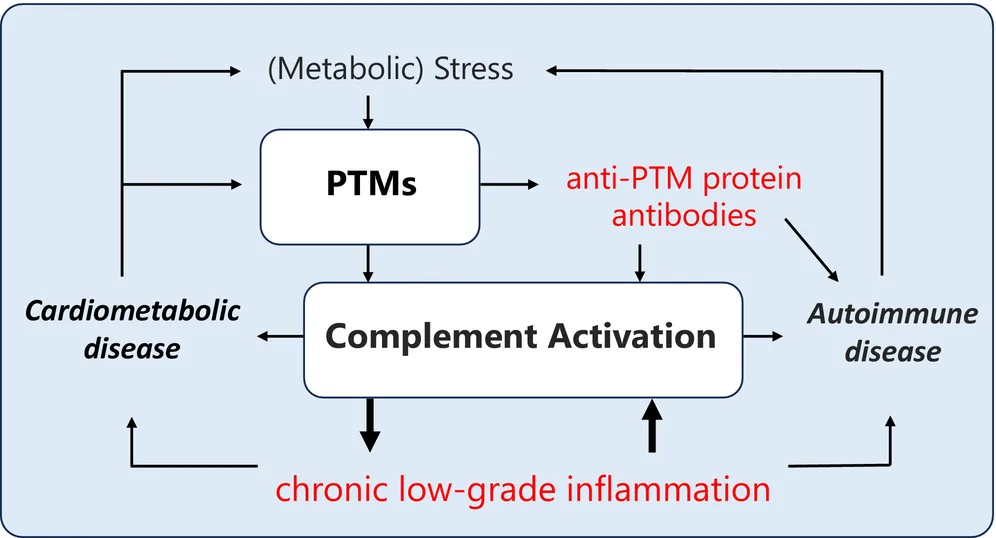
Proposed crosstalk between post‐translationally modified (PTM) proteins, complement and inflammation contributing to the development of cardiometabolic and autoimmune diseases. Non‐enzymatically induced PTMs are often generated via factors present in the intra‐ and extracellular milieu, including endoplasmic reticulum stress, mitochondrial dysfunction and oxidative stress, for example, as a consequence of nutrient excess which may overcharge the intrinsic metabolic capacity of cells. These PTM‐proteins may serve as a driver of chronic low‐level complement activation. On the one hand because complement binds to, and is activated on, the PTM protein, but additionally because PTM formation on complement regulators may modulate the complement response, in general. Moreover, the presence of PTMs induces the production of anti‐modified protein antibodies (AMPA) which may further enhance complement activation. Chronic low‐grade complement activation by PTMs and PTM antibodies induces a low‐grade inflammatory response. A major effector of complement‐induced low‐grade inflammation is inflammasome activation. Complement and inflammasome activation are mutual amplifiers via bidirectional crosstalk. Local and systemic complement activation combined with an inflammatory response contributes to many non‐communicable diseases, including cardiometabolic diseases such as cardiovascular disease and Type 2 diabetes mellitus, autoimmune diseases such as rheumatoid arthritis as well as their comorbidities.

Post‐translational modification of proteins can change protein function and structure. Some PTM‐proteins have the capacity to activate complement, either directly by binding to complement recognition molecules or indirectly via the binding of anti‐PTM autoantibodies. Complement activation that is driven by PTM‐proteins and anti‐PTM antibodies stimulates chronic inflammation, which is a main driver of cardiometabolic and autoimmune diseases (Figure 4). A decrease in the levels of specific PTM‐proteins, which may be achieved by changing lifestyle factors and/or environmental exposure as well as via specific PTM‐targeted complement inhibition, may attenuate PTM‐driven inflammation and thereby reduce the onset of cardiometabolic and autoimmune diseases and their associated comorbidities.

## Conflicts of Interest

L.A.T. is mentioned as an inventor on a patent describing the detection of anti‐CarP antibodies in rheumatoid arthritis and on a patent describing local targeted complement inhibition using bispecific antibodies.

## Data Availability

Data sharing not applicable to this article as no datasets were generated or analysed during the current study.

## References

[1] D. C. Mastellos , G. Hajishengallis , and J. D. Lambris , “A Guide to Complement Biology, Pathology and Therapeutic Opportunity,” Nature Reviews. Immunology 24, no. 2 (2024): 118–141. 10.1038/s41577-023-00926-1 37670180

[2] A. J. Nauta , L. A. Trouw , M. R. Daha , et al., “Direct Binding of C1q to Apoptotic Cells and Cell Blebs Induces Complement Activation,” European Journal of Immunology 32, no. 6 (2002): 1726–1736. 12115656 10.1002/1521-4141(200206)32:6<1726::AID-IMMU1726>3.0.CO;2-R

[3] R. Lubbers , M. F. van Essen , C. van Kooten , and L. A. Trouw , “Production of Complement Components by Cells of the Immune System,” Clinical and Experimental Immunology 188, no. 2 (2017): 183–194. 28249350 10.1111/cei.12952 PMC5383442

[4] L. A. Trouw and M. R. Daha , “Role of Complement in Innate Immunity and Host Defense,” Immunology Letters 138, no. 1 (2011): 35–37. 21333684 10.1016/j.imlet.2011.02.014

[5] A. P. Sjoberg , L. A. Trouw , and A. M. Blom , “Complement Activation and Inhibition: A Delicate Balance,” Trends in Immunology 30, no. 2 (2009): 83–90. 19144569 10.1016/j.it.2008.11.003

[6] T. Ebert , N. Tran , L. Schurgers , P. Stenvinkel , and P. G. Shiels , “Ageing‐Oxidative Stress, PTMs and Disease,” Molecular Aspects of Medicine 86 (2022): 101099. 35689974 10.1016/j.mam.2022.101099

[7] E. Q. Jennings , K. S. Fritz , and J. J. Galligan , “Biochemical Genesis of Enzymatic and Non‐Enzymatic Post‐Translational Modifications,” Molecular Aspects of Medicine 86 (2022): 101053. 34838336 10.1016/j.mam.2021.101053 PMC9126990

[8] V. J. Victorino , A. L. Mencalha , and C. Panis , “Post‐Translational Modifications Disclose a Dual Role for Redox Stress in Cardiovascular Pathophysiology,” Life Sciences 129 (2015): 42–47. 25433127 10.1016/j.lfs.2014.11.008

[9] K. H. Gaens , C. D. Stehouwer , and C. G. Schalkwijk , “Advanced Glycation Endproducts and Its Receptor for Advanced Glycation Endproducts in Obesity,” Current Opinion in Lipidology 24, no. 1 (2013): 4–11. 23298958 10.1097/MOL.0b013e32835aea13

[10] V. M. Monnier and A. Cerami , “Nonenzymatic Browning In Vivo: Possible Process for Aging of Long‐Lived Proteins,” Science 211, no. 4481 (1981): 491–493. 6779377 10.1126/science.6779377

[11] L. Gorisse , C. Pietrement , V. Vuiblet , et al., “Protein Carbamylation is a Hallmark of Aging,” Proceedings of the National Academy of Sciences of the United States of America 113, no. 5 (2016): 1191–1196. 26712018 10.1073/pnas.1517096113 PMC4747743

[12] C. G. Schalkwijk and C. D. A. Stehouwer , “Methylglyoxal, a Highly Reactive Dicarbonyl Compound, in Diabetes, Its Vascular Complications, and Other Age‐Related Diseases,” Physiological Reviews 100, no. 1 (2020): 407–461. 31539311 10.1152/physrev.00001.2019

[13] S. Zakhari , “Overview: How is Alcohol Metabolized by the Body?,” Alcohol Research & Health 29, no. 4 (2006): 245–254. 17718403 PMC6527027

[14] L. M. Kraus , A. J. Elberger , C. R. Handorf , M. J. Pabst , and A. P. Kraus, Jr. , “Urea‐Derived Cyanate Forms Epsilon‐Amino‐Carbamoyl‐Lysine (Homocitrulline) in Leukocyte Proteins in Patients With End‐Stage Renal Disease on Peritoneal Dialysis,” Journal of Laboratory and Clinical Medicine 123, no. 6 (1994): 882–891. 8201267

[15] M. Singh , A. Kapoor , and A. Bhatnagar , “Oxidative and Reductive Metabolism of Lipid‐Peroxidation Derived Carbonyls,” Chemico‐Biological Interactions 234 (2015): 261–273. 25559856 10.1016/j.cbi.2014.12.028 PMC4414726

[16] F. W. Chaplen , “Incidence and Potential Implications of the Toxic Metabolite Methylglyoxal in Cell Culture: A Review,” Cytotechnology 26, no. 3 (1998): 173–183. 22358615 10.1023/A:1007953628840 PMC3449548

[17] S. Delanghe , J. R. Delanghe , R. Speeckaert , W. Van Biesen , and M. M. Speeckaert , “Mechanisms and Consequences of Carbamoylation,” Nature Reviews. Nephrology 13, no. 9 (2017): 580–593. 28757635 10.1038/nrneph.2017.103

[18] D. R. Sell and V. M. Monnier , “Molecular Basis of Arterial Stiffening: Role of Glycation—a Mini‐Review,” Gerontology 58, no. 3 (2012): 227–237. 22222677 10.1159/000334668

[19] Z. Wang , S. J. Nicholls , E. R. Rodriguez , et al., “Protein Carbamylation Links Inflammation, Smoking, Uremia and Atherogenesis,” Nature Medicine 13, no. 10 (2007): 1176–1184. 10.1038/nm1637 17828273

[20] X. Gallart‐Palau , L. M. Tan , A. Serra , et al., “Degenerative Protein Modifications in the Aging Vasculature and Central Nervous System: A Problem Shared Is Not Always Halved,” Ageing Research Reviews 53 (2019): 100909. 31116994 10.1016/j.arr.2019.100909

[21] Z. Zhang , Y. Wang , T. Li , and H. Wang , “NETosis in Myocardial Ischemia‐Reperfusion Injury: From Mechanisms to Therapies (Review),” Biomedical Reports 23, no. 1 (2025): 113. 40420974 10.3892/br.2025.1991 PMC12105085

[22] M. Akagawa , “Protein Carbonylation: Molecular Mechanisms, Biological Implications, and Analytical Approaches,” Free Radical Research 55, no. 4 (2021): 307–320. 33183115 10.1080/10715762.2020.1851027

[23] M. Hellwig , P. Diel , G. Eisenbrand , et al., “Dietary Glycation Compounds‐Implications for Human Health,” Critical Reviews in Toxicology 54, no. 8 (2024): 485–617. 39150724 10.1080/10408444.2024.2362985

[24] A. M. Pickering , A. L. Koop , C. Y. Teoh , G. Ermak , T. Grune , and K. J. Davies , “The Immunoproteasome, the 20S Proteasome and the Pa28alphabeta Proteasome Regulator Are Oxidative‐Stress‐Adaptive Proteolytic Complexes,” Biochemical Journal 432, no. 3 (2010): 585–594. 20919990 10.1042/BJ20100878 PMC3133595

[25] D. T. Antoniak , M. J. Duryee , T. R. Mikuls , G. M. Thiele , and D. R. Anderson , “Aldehyde‐Modified Proteins as Mediators of Early Inflammation in Atherosclerotic Disease,” Free Radical Biology & Medicine 89 (2015): 409–418. 26432980 10.1016/j.freeradbiomed.2015.09.003

[26] M. J. Duryee , L. W. Klassen , C. S. Schaffert , et al., “Malondialdehyde‐Acetaldehyde Adduct is the Dominant Epitope After MDA Modification of Proteins in Atherosclerosis,” Free Radical Biology & Medicine 49, no. 10 (2010): 1480–1486. 20696236 10.1016/j.freeradbiomed.2010.08.001 PMC2952714

[27] K. Lim and S. Kalim , “The Role of Nonenzymatic Post‐Translational Protein Modifications in Uremic Vascular Calcification,” Advances in Chronic Kidney Disease 26, no. 6 (2019): 427–436. 31831121 10.1053/j.ackd.2019.10.001 PMC6993873

[28] F. H. Verbrugge , W. H. Tang , and S. L. Hazen , “Protein Carbamylation and Cardiovascular Disease,” Kidney International 88, no. 3 (2015): 474–478. 26061545 10.1038/ki.2015.166 PMC4556561

[29] A. Awwad , M. Tang , and S. Kalim , “Translating Carbamylation Biology Into Precision Medicine for Kidney Disease,” Clinical Journal of the American Society of Nephrology (2026). 10.2215/CJN.0000001061 41842960

[30] M. J. Duryee , N. Aripova , C. D. Hunter , et al., “A Novel Reactive Aldehyde Species Inhibitor Prevents the Deleterious Effects of Ethanol in an Animal Model of Alcoholic Liver Disease,” International Immunopharmacology 113, no. Pt A (2022): 109400. 36461583 10.1016/j.intimp.2022.109400

[31] M. J. Duryee , L. W. Klassen , T. L. Freeman , M. S. Willis , D. J. Tuma , and G. M. Thiele , “Chronic Ethanol Consumption Impairs Receptor‐Mediated Endocytosis of MAA‐Modified Albumin by Liver Endothelial Cells,” Biochemical Pharmacology 66, no. 6 (2003): 1045–1054. 12963492 10.1016/s0006-2952(03)00416-7

[32] G. M. Thiele , M. J. Duryee , M. S. Willis , et al., “Malondialdehyde‐Acetaldehyde (MAA) Modified Proteins Induce Pro‐Inflammatory and Pro‐Fibrotic Responses by Liver Endothelial Cells,” Comparative Hepatology 3, no. Suppl 1 (2004): S25. 14960177 10.1186/1476-5926-2-S1-S25 PMC2410244

[33] G. J. Pruijn , “Citrullination and Carbamylation in the Pathophysiology of Rheumatoid Arthritis,” Frontiers in Immunology 6 (2015): 192. 25964785 10.3389/fimmu.2015.00192 PMC4410602

[34] M. K. Verheul , G. M. C. Janssen , A. de Ru , et al., “Mass‐Spectrometric Identification of Carbamylated Proteins Present in the Joints of Rheumatoid Arthritis Patients and Controls,” Clinical and Experimental Rheumatology 39, no. 3 (2021): 570–577. 32896247

[35] M. J. Duryee , D. L. Clemens , P. J. Opperman , et al., “Malondialdehyde‐Acetaldehyde Modified (MAA) Proteins Differentially Effect the Inflammatory Response in Macrophage, Endothelial Cells and Animal Models of Cardiovascular Disease,” International Journal of Molecular Sciences 22, no. 23 (2021): 12948. 34884754 10.3390/ijms222312948 PMC8657968

[36] A. V. Poznyak , V. N. Sukhorukov , R. Surkova , N. A. Orekhov , and A. N. Orekhov , “Glycation of LDL: AGEs, Impact on Lipoprotein Function, and Involvement in Atherosclerosis,” Frontiers in Cardiovascular Medicine 10 (2023): 1094188. 36760567 10.3389/fcvm.2023.1094188 PMC9904536

[37] C. Xu , J. Rodriguez‐Carrio , Y. Xie , et al., “Post‐Translational Modifications of Lipoproteins: Emerging Players Linking Inflammation and Cardiovascular Disease in Rheumatoid Arthritis‐A Narrative Review,” International Journal of Molecular Sciences 26, no. 17 (2025): 8514. 40943434 10.3390/ijms26178514 PMC12428818

[38] N. Niyonzima , J. Rahman , N. Kunz , et al., “Mitochondrial C5aR1 Activity in Macrophages Controls IL‐1beta Production Underlying Sterile Inflammation,” Science Immunology 6, no. 66 (2021): eabf2489. 34932384 10.1126/sciimmunol.abf2489 PMC8902698

[39] S. Accacha , I. Voloshyna , L. J. Kasselman , et al., “Plasma From Type 1 Diabetes Patients Promotes Pro‐Atherogenic Cholesterol Transport in Human Macrophages,” Journal of Investigative Medicine 73, no. 2 (2025): 183–192. 39417428 10.1177/10815589241296025

[40] E. O. Apostolov , S. V. Shah , D. Ray , and A. G. Basnakian , “Scavenger Receptors of Endothelial Cells Mediate the Uptake and Cellular Proatherogenic Effects of Carbamylated LDL,” Arteriosclerosis, Thrombosis, and Vascular Biology 29, no. 10 (2009): 1622–1630. 19696406 10.1161/ATVBAHA.109.189795 PMC5075391

[41] M. Sapkota , J. M. DeVasure , K. K. Kharbanda , and T. A. Wyatt , “Malondialdehyde‐Acetaldehyde (MAA) Adducted Surfactant Protein Induced Lung Inflammation is Mediated Through Scavenger Receptor a (SR‐A1),” Respiratory Research 18, no. 1 (2017): 36. 28193223 10.1186/s12931-017-0517-x PMC5307820

[42] G. M. Thiele , M. J. Duryee , C. D. Hunter , et al., “Immunogenic and Inflammatory Responses to Citrullinated Proteins Are Enhanced Following Modification With Malondialdehyde‐Acetaldehyde Adducts,” International Immunopharmacology 83 (2020): 106433. 32224441 10.1016/j.intimp.2020.106433 PMC7250734

[43] S. Park , S. J. Yoon , H. J. Tae , and C. Y. Shim , “RAGE and Cardiovascular Disease,” Front Biosci (Landmark Ed) 16, no. 2 (2011): 486–497. 21196183 10.2741/3700

[44] D. A. Chistiakov , A. A. Melnichenko , V. A. Myasoedova , A. V. Grechko , and A. N. Orekhov , “Mechanisms of Foam Cell Formation in Atherosclerosis,” Journal of Molecular Medicine (Berlin, Germany) 95, no. 11 (2017): 1153–1165. 28785870 10.1007/s00109-017-1575-8

[45] J. Sokolove , X. Zhao , P. E. Chandra , and W. H. Robinson , “Immune Complexes Containing Citrullinated Fibrinogen Costimulate Macrophages via Toll‐Like Receptor 4 and Fcgamma Receptor,” Arthritis & Rheumatism 63, no. 1 (2011): 53–62. 20954191 10.1002/art.30081 PMC3015008

[46] B. Raposo , L. Klareskog , W. H. Robinson , V. Malmstrom , and C. Gronwall , “The Peculiar Features, Diversity and Impact of Citrulline‐Reactive Autoantibodies,” Nature Reviews Rheumatology 20, no. 7 (2024): 399–416. 38858604 10.1038/s41584-024-01124-6

[47] G. Cambridge , J. Acharya , J. A. Cooper , J. C. Edwards , and S. E. Humphries , “Antibodies to Citrullinated Peptides and Risk of Coronary Heart Disease,” Atherosclerosis 228, no. 1 (2013): 243–246. 23474125 10.1016/j.atherosclerosis.2013.02.009

[48] E. Korca , V. Piskovatska , J. Borgermann , A. Navarrete Santos , and A. Simm , “Circulating Antibodies Against Age‐Modified Proteins in Patients With Coronary Atherosclerosis,” Scientific Reports 10, no. 1 (2020): 17105. 33051525 10.1038/s41598-020-73877-5 PMC7553914

[49] K. Kostov and A. Blazhev , “Elevated IgG and IgM Autoantibodies to Advanced Glycation End Products of Vascular Elastin in Hypertensive Patients With Type 2 Diabetes: Relevance to Disease Initiation and Progression,” Pathophysiology 29, no. 3 (2022): 426–434. 35997390 10.3390/pathophysiology29030034 PMC9396981

[50] M. F. Lopes‐Virella , K. J. Hunt , N. L. Baker , and G. Virella , “High Levels of AGE‐LDL, and of IgG Antibodies Reacting With MDA‐Lysine Epitopes Expressed by oxLDL and MDA‐LDL in Circulating Immune Complexes Predict Macroalbuminuria in Patients With Type 2 Diabetes,” Journal of Diabetes and Its Complications 30, no. 4 (2016): 693–699. 26861948 10.1016/j.jdiacomp.2016.01.012

[51] L. A. Trouw , T. Rispens , and R. E. M. Toes , “Beyond Citrullination: Other Post‐Translational Protein Modifications in Rheumatoid Arthritis,” Nature Reviews Rheumatology 13, no. 6 (2017): 331–339. 28275265 10.1038/nrrheum.2017.15

[52] A. S. B. Kampstra , J. S. Dekkers , M. Volkov , et al., “Different Classes of Anti‐Modified Protein Antibodies Are Induced on Exposure to Antigens Expressing Only One Type of Modification,” Annals of the Rheumatic Diseases 78, no. 7 (2019): 908–916. 31151934 10.1136/annrheumdis-2018-214950

[53] K. A. Sveen , K. Bech Holte , M. Svanteson , et al., “Autoantibodies Against Methylglyoxal‐Modified Apolipoprotein B100 and ApoB100 Peptide Are Associated With Less Coronary Artery Atherosclerosis and Retinopathy in Long‐Term Type 1 Diabetes,” Diabetes Care 44, no. 6 (2021): 1402–1409. 33858856 10.2337/dc20-2089 PMC8247486

[54] G. A. Schellekens , B. A. de Jong , F. H. van den Hoogen , L. B. van de Putte , and W. J. van Venrooij , “Citrulline is an Essential Constituent of Antigenic Determinants Recognized by Rheumatoid Arthritis‐Specific Autoantibodies,” Journal of Clinical Investigation 101, no. 1 (1998): 273–281. 9421490 10.1172/JCI1316 PMC508564

[55] J. Shi , R. Knevel , P. Suwannalai , et al., “Autoantibodies Recognizing Carbamylated Proteins Are Present in Sera of Patients With Rheumatoid Arthritis and Predict Joint Damage,” Proceedings of the National Academy of Sciences of the United States of America 108, no. 42 (2011): 17372–17377. 21987802 10.1073/pnas.1114465108 PMC3198314

[56] G. M. Thiele , M. J. Duryee , D. R. Anderson , et al., “Malondialdehyde‐Acetaldehyde Adducts and Anti‐Malondialdehyde‐Acetaldehyde Antibodies in Rheumatoid Arthritis,” Arthritis & Rhematology 67, no. 3 (2015): 645–655. 10.1002/art.38969 PMC5469548 25417811

[57] M. D. van den Beukel , R. C. Monahan , N. V. Borggreven , T. W. J. Huizinga , M. G. Steup‐Beekman , and L. A. Trouw , “Malondialdehyde‐Acetaldehyde Antibodies Occur in Systemic Lupus Erythematosus and Associate Specifically With Neuropsychiatric Involvement,” European Journal of Immunology 51 (2021): 268.33544898

[58] M. D. van den Beukel , T. J. van Wesemael , A. T. W. Hoogslag , et al., “Antibodies Against Advanced Glycation End‐Products and Malondialdehyde‐Acetaldehyde Adducts Identify a New Specific Subgroup of Hitherto Patients With Seronegative Arthritis With a Distinct Clinical Phenotype and an HLA Class II Association,” RMD Open 9, no. 4 (2023): e003480. 38053459 10.1136/rmdopen-2023-003480 PMC10693877

[59] Z. Turk , S. Ljubic , N. Turk , and B. Benko , “Detection of Autoantibodies Against Advanced Glycation Endproducts and AGE‐Immune Complexes in Serum of Patients With Diabetes Mellitus,” Clinica Chimica Acta 303, no. 1–2 (2001): 105–115. 10.1016/s0009-8981(00)00389-2 11163030

[60] M. D. van den Beukel , A. E. C. Stoelinga , A. J. van der Meer , et al., “Antibodies Against Multiple Post‐Translationally Modified Proteins Aid in Diagnosis of Autoimmune Hepatitis and Associate With Complete Biochemical Response to Treatment,” Front Med (Lausanne) 10 (2023): 1195747. 37564051 10.3389/fmed.2023.1195747 PMC10411548

[61] J. S. Nijjar , F. R. Morton , H. Bang , et al., “The Impact of Autoantibodies Against Citrullinated, Carbamylated, and Acetylated Peptides on Radiographic Progression in Patients With New‐Onset Rheumatoid Arthritis: An Observational Cohort Study,” Lancet Rheumatology 3, no. 4 (2021): e284–e293. 34604794 10.1016/S2665-9913(20)30381-7 PMC7611758

[62] A. H. van der Helm‐van Mil , K. N. Verpoort , F. C. Breedveld , R. E. Toes , and T. W. Huizinga , “Antibodies to Citrullinated Proteins and Differences in Clinical Progression of Rheumatoid Arthritis,” Arthritis Research & Therapy 7, no. 5 (2005): R949–R958. 16207336 10.1186/ar1767 PMC1257421

[63] C. Clavel , L. Nogueira , L. Laurent , et al., “Induction of Macrophage Secretion of Tumor Necrosis Factor Alpha Through Fcgamma Receptor IIa Engagement by Rheumatoid Arthritis‐Specific Autoantibodies to Citrullinated Proteins Complexed With Fibrinogen,” Arthritis and Rheumatism 58, no. 3 (2008): 678–688. 18311806 10.1002/art.23284

[64] L. A. Trouw , E. M. Haisma , E. W. N. Levarht , et al., “Anti‐Cyclic Citrullinated Peptide Antibodies From Rheumatoid Arthritis Patients Activate Complement via Both the Classical and Alternative Pathways,” Arthritis and Rheumatism 60, no. 7 (2009): 1923–1931. 19565507 10.1002/art.24622

[65] R. W. Gan , L. A. Trouw , J. Shi , et al., “Anti‐Carbamylated Protein Antibodies Are Present Prior to Rheumatoid Arthritis and Are Associated With Its Future Diagnosis,” Journal of Rheumatology 42, no. 4 (2015): 572–579. 25593232 10.3899/jrheum.140767 PMC4383711

[66] T. R. Mikuls , J. Edison , E. Meeshaw , et al., “Autoantibodies to Malondialdehyde‐Acetaldehyde Are Detected Prior to Rheumatoid Arthritis Diagnosis and After Other Disease Specific Autoantibodies,” Arthritis & Rhematology 72, no. 12 (2020): 2025–2029. 10.1002/art.41424 PMC7722146 32621635

[67] J. Shi , L. A. van de Stadt , E. W. N. Levarht , et al., “Anti‐Carbamylated Protein Antibodies (Anti‐CarP) Precede The Onset Of Rheumatoid Arthritis,” Arthritis and Rheumatism 65 (2013): S391. 10.1002/art.37830 23279976

[68] R. Biersteker , A. Athanasiadou , S. van der Meulen , et al., “Insights Into the Detection of AMPA Cross‐Reactivity: Comparing Cyclic Peptide‐ to Protein‐Based Assays,” Arthritis Research & Therapy 27, no. 1 (2025): 138. 40624574 10.1186/s13075-025-03591-y PMC12232869

[69] P. Sahlstrom , M. Hansson , J. Steen , et al., “Different Hierarchies of Anti‐Modified Protein Autoantibody Reactivities in Rheumatoid Arthritis,” Arthritis and Rheumatology 72, no. 10 (2020): 1643–1657. 32501655 10.1002/art.41385

[70] V. Joshua , M. Loberg Haarhaus , A. Hensvold , et al., “Rheumatoid Arthritis‐Specific Autoimmunity in the Lung Before and at the Onset of Disease,” Arthritis and Rheumatology 75, no. 11 (2023): 1910–1922. 37192126 10.1002/art.42549

[71] M. Volkov , A. S. B. Kampstra , K. A. van Schie , et al., “Evolution of Anti‐Modified Protein Antibody Responses Can be Driven by Consecutive Exposure to Different Post‐Translational Modifications,” Arthritis Research & Therapy 23, no. 1 (2021): 298. 34876234 10.1186/s13075-021-02687-5 PMC8653599

[72] M. Chikazawa , T. Shibata , Y. Hatasa , et al., “Identification of C1q as a Binding Protein for Advanced Glycation End Products,” Biochemistry 55, no. 3 (2016): 435–446. 26731343 10.1021/acs.biochem.5b00777

[73] P. D. Frederiksen , S. Thiel , C. B. Larsen , and J. C. Jensenius , “M‐Ficolin, an Innate Immune Defence Molecule, Binds Patterns of Acetyl Groups and Activates Complement,” Scandinavian Journal of Immunology 62, no. 5 (2005): 462–473. 16305643 10.1111/j.1365-3083.2005.01685.x

[74] E. Hein , C. Honore , M. O. Skjoedt , L. Munthe‐Fog , T. Hummelshoj , and P. Garred , “Functional Analysis of Ficolin‐3 Mediated Complement Activation,” PLoS One 5, no. 11 (2010): e15443. 21085669 10.1371/journal.pone.0015443 PMC2978102

[75] B. Klop , P. van der Pol , R. van Bruggen , et al., “Differential Complement Activation Pathways Promote C3b Deposition on Native and Acetylated LDL Thereby Inducing Lipoprotein Binding to the Complement Receptor 1,” Journal of Biological Chemistry 289, no. 51 (2014): 35421–35430. 25349208 10.1074/jbc.M114.573840 PMC4271227

[76] A. Krarup , S. Thiel , A. Hansen , T. Fujita , and J. C. Jensenius , “L‐Ficolin Is a Pattern Recognition Molecule Specific for Acetyl Groups,” Journal of Biological Chemistry 279, no. 46 (2004): 47513–47519. 15331601 10.1074/jbc.M407161200

[77] M. D. van den Beukel , L. Zhang , S. van der Meulen , et al., “Post‐Translationally Modified Proteins Bind and Activate Complement With Implications for Cellular Uptake and Autoantibody Formation,” Journal of Autoimmunity 155 (2025): 103444. 40554855 10.1016/j.jaut.2025.103444

[78] P. W. Dempsey , M. E. Allison , S. Akkaraju , C. C. Goodnow , and D. T. Fearon , “C3d of Complement as a Molecular Adjuvant: Bridging Innate and Acquired Immunity,” Science 271, no. 5247 (1996): 348–350. 8553069 10.1126/science.271.5247.348

[79] M. K. Verheul , P. A. van Veelen , M. A. M. van Delft , et al., “Pitfalls in the Detection of Citrullination and Carbamylation,” Autoimmunity Reviews 17, no. 2 (2018): 136–141. 29203292 10.1016/j.autrev.2017.11.017

[80] T. Senshu , T. Sato , T. Inoue , K. Akiyama , and H. Asaga , “Detection of Citrulline Residues in Deiminated Proteins on Polyvinylidene Difluoride Membrane,” Analytical Biochemistry 203, no. 1 (1992): 94–100. 1524220 10.1016/0003-2697(92)90047-b

[81] J. Shi , A. Willemze , G. M. C. Janssen , et al., “Recognition of Citrullinated and Carbamylated Proteins by Human Antibodies: Specificity, Cross‐Reactivity and the ‘AMC‐Senshu’ Method,” Annals of the Rheumatic Diseases 72, no. 1 (2013): 148–150. 22843489 10.1136/annrheumdis-2012-201559

[82] D. E. Linnea Lindelöf , K. Ekdahl , P. Garred , B. Nilsson , and O. Eriksson , “Recognition of Post‐Translational Lysine Modifications by the Lectin Pathway Initiator FICOLIN‐3. Abstracts EMCHD,” Molecular Immunology (2022).

[83] M. Veneskoski , S. P. Turunen , O. Kummu , et al., “Specific Recognition of Malondialdehyde and Malondialdehyde Acetaldehyde Adducts on Oxidized LDL and Apoptotic Cells by Complement Anaphylatoxin C3a,” Free Radical Biology and Medicine 51, no. 4 (2011): 834–843. 21683785 10.1016/j.freeradbiomed.2011.05.029

[84] L. Alic , C. J. Binder , and N. Papac‐Milicevic , “The OSE Complotype and Its Clinical Potential,” Frontiers in Immunology 13 (2022): 1010893. 36248824 10.3389/fimmu.2022.1010893 PMC9561429

[85] R. B. Rudnick , Q. Chen , E. D. Stea , et al., “FHR5 Binds to Laminins, Uses Separate C3b and Surface‐Binding Sites, and Activates Complement on Malondialdehyde‐Acetaldehyde Surfaces,” Journal of Immunology 200, no. 7 (2018): 2280–2290. 10.4049/jimmunol.1701641 29483359

[86] Y. Xin , E. Hertle , C. J. H. van der Kallen , C. G. Schalkwijk , C. D. A. Stehouwer , and M. M. J. van Greevenbroek , “Associations of Dicarbonyl Stress With Complement Activation: The CODAM Study,” Diabetologia 63, no. 5 (2020): 1032–1042. 31993713 10.1007/s00125-020-05098-4 PMC7145776

[87] M. G. Kiss , N. Papac‐Milicevic , F. Porsch , et al., “Cell‐Autonomous Regulation of Complement C3 by Factor H Limits Macrophage Efferocytosis and Exacerbates Atherosclerosis,” Immunity 56, no. 8 (2023): 1809–1824.e10. 37499656 10.1016/j.immuni.2023.06.026 PMC10529786

[88] S. Hyvarinen , K. Uchida , M. Varjosalo , R. Jokela , and T. S. Jokiranta , “Recognition of Malondialdehyde‐Modified Proteins by the C Terminus of Complement Factor H Is Mediated via the Polyanion Binding Site and Impaired by Mutations Found in Atypical Hemolytic Uremic Syndrome,” Journal of Biological Chemistry 289, no. 7 (2014): 4295–4306. 24344133 10.1074/jbc.M113.527416 PMC3924292

[89] D. Weismann , K. Hartvigsen , N. Lauer , et al., “Complement Factor H Binds Malondialdehyde Epitopes and Protects From Oxidative Stress,” Nature 478, no. 7367 (2011): 76–81. 21979047 10.1038/nature10449 PMC4826616

[90] L. A. Trouw , A. A. Bengtsson , K. A. Gelderman , B. Dahlback , G. Sturfelt , and A. M. Blom , “C4b‐Binding Protein and Factor h Compensate for the Loss of Membrane‐Bound Complement Inhibitors to Protect Apoptotic Cells Against Excessive Complement Attack,” Journal of Biological Chemistry 282, no. 39 (2007): 28540–28548. 17699521 10.1074/jbc.M704354200

[91] M. Gaya da Costa , F. Poppelaars , C. van Kooten , et al., “Age and Sex‐Associated Changes of Complement Activity and Complement Levels in a Healthy Caucasian Population,” Frontiers in Immunology 9 (2018): 2664. 30515158 10.3389/fimmu.2018.02664 PMC6255829

[92] C. L. Harris , M. Heurich , S. Rodriguez de Cordoba , and B. P. Morgan , “The Complotype: Dictating Risk for Inflammation and Infection,” Trends in Immunology 33, no. 10 (2012): 513–521. 22749446 10.1016/j.it.2012.06.001 PMC3460238

[93] S. Jaisson , I. Kazes , A. Desmons , et al., “Homocitrulline as Marker of Protein Carbamylation in Hemodialyzed Patients,” Clinica Chimica Acta 460 (2016): 5–10. 10.1016/j.cca.2016.06.009 27302313

[94] F. Poppelaars , B. Faria , M. Gaya da Costa , et al., “The Complement System in Dialysis: A Forgotten Story?,” Frontiers in Immunology 9 (2018): 71. 29422906 10.3389/fimmu.2018.00071 PMC5788899

[95] R. Biersteker , L. Abendstein , S. van de Bovenkamp , et al., “Modulation of Immunoglobulin G Oligomerization by Variable Domain Glycans: A Mechanism to Regulate Complement Activation,” PNAS Nexus 4, no. 7 (2025): pgaf216. 40735689 10.1093/pnasnexus/pgaf216 PMC12305304

[96] H. Wang , S. Nugteren , R. J. Groenland , et al., “Development of Bispecific Antibodies That Drive Selective Targeted Local Complement Inhibition on Rheumatoid Arthritis‐Related Antigens,” Annals of the Rheumatic Diseases 84, no. 10 (2025): 1641–1648. 40683829 10.1016/j.ard.2025.06.2130

[97] D. Rajcic , B. P. da Silva , T. Baranovskyi , et al., “IgM Antibodies Targeting Malondialdehyde Promote Complement‐Mediated Liver Injury in Alcohol‐Related Liver Disease,” Liver International 45, no. 10 (2025): e70356. 40960284 10.1111/liv.70356 PMC12442528

[98] P. Suwannalai , K. Britsemmer , R. Knevel , et al., “Low‐Avidity Anticitrullinated Protein Antibodies (ACPA) Are Associated With a Higher Rate of Joint Destruction in Rheumatoid Arthritis,” Annals of the Rheumatic Diseases 73, no. 1 (2014): 270–276. 23463689 10.1136/annrheumdis-2012-202615

[99] D. R. Schultz and P. I. Arnold , “Cyanate as an Inactivator of Complement Proteins,” Journal of Immunology 115, no. 6 (1975): 1558–1565. 1184967

[100] X. Qin , A. Goldfine , N. Krumrei , et al., “Glycation Inactivation of the Complement Regulatory Protein CD59: A Possible Role in the Pathogenesis of the Vascular Complications of Human Diabetes,” Diabetes 53, no. 10 (2004): 2653–2661. 15448097 10.2337/diabetes.53.10.2653

[101] R. Fluckiger , E. Cocuzzi , R. H. Nagaraj , M. Shoham , T. S. Kern , and M. E. Medof , “DAF in Diabetic Patients Is Subject to Glycation/Inactivation at Its Active Site Residues,” Molecular Immunology 93 (2018): 246–252. 28886871 10.1016/j.molimm.2017.06.036 PMC5884443

[102] K. Hess , S. H. Alzahrani , M. Mathai , et al., “A Novel Mechanism for Hypofibrinolysis in Diabetes: The Role of Complement C3,” Diabetologia 55, no. 4 (2012): 1103–1113. 21918806 10.1007/s00125-011-2301-7

[103] K. Hess , S. H. Alzahrani , J. F. Price , et al., “Hypofibrinolysis in Type 2 Diabetes: The Role of the Inflammatory Pathway and Complement C3,” Diabetologia 57, no. 8 (2014): 1737–1741. 24838681 10.1007/s00125-014-3267-z

[104] M. Zamboni , N. Nori , A. Brunelli , and E. Zoico , “How Does Adipose Tissue Contribute to Inflammageing?,” Experimental Gerontology 143 (2021): 111162. 33253807 10.1016/j.exger.2020.111162

[105] S. de Ferranti and D. Mozaffarian , “The Perfect Storm: Obesity, Adipocyte Dysfunction, and Metabolic Consequences,” Clinical Chemistry 54, no. 6 (2008): 945–955. 18436717 10.1373/clinchem.2007.100156

[106] X. Xu , Y. Pang , and X. Fan , “Mitochondria in Oxidative Stress, Inflammation and Aging: From Mechanisms to Therapeutic Advances,” Signal Transduction and Targeted Therapy 10, no. 1 (2025): 190. 40500258 10.1038/s41392-025-02253-4 PMC12159213

[107] I. G. Lempesis , R. L. J. van Meijel , K. N. Manolopoulos , and G. H. Goossens , “Oxygenation of Adipose Tissue: A Human Perspective,” Acta Physiologica (Oxford) 228, no. 1 (2020): e13298. 10.1111/apha.13298 PMC6916558 31077538

[108] E. Hertle , M. M. van Greevenbroek , I. C. Arts , et al., “Complement Activation Products C5a and sC5b‐9 Are Associated With Low‐Grade Inflammation and Endothelial Dysfunction, But not With Atherosclerosis in a Cross‐Sectional Analysis: The CODAM Study,” International Journal of Cardiology 174, no. 2 (2014): 400–403. 24794956 10.1016/j.ijcard.2014.04.057

[109] E. Hertle , C. D. Stehouwer , and M. M. van Greevenbroek , “The Complement System in Human Cardiometabolic Disease,” Molecular Immunology 61, no. 2 (2014): 135–148. 25017306 10.1016/j.molimm.2014.06.031

[110] Z. Guo , X. Fan , J. Yao , S. Tomlinson , G. Yuan , and S. He , “The Role of Complement in Nonalcoholic Fatty Liver Disease,” Frontiers in Immunology 13 (2022): 1017467. 36248852 10.3389/fimmu.2022.1017467 PMC9562907

[111] H. Tilg and M. Effenberger , “From NAFLD to MAFLD: When Pathophysiology Succeeds,” Nature Reviews. Gastroenterology & Hepatology 17, no. 7 (2020): 387–388. 10.1038/s41575-020-0316-6 32461575

[112] M. Guldan , L. Ozbek , S. M. Abdel‐Rahman , et al., “Targeting Visceral and Ectopic Adiposity: Pharmacological and Surgical Interventions Beyond Global Weight Loss,” Diabetes, Obesity & Metabolism 27, no. 11 (2025): 6116–6138. 10.1111/dom.70019 40798892

[113] J. G. Letarouilly , R. M. Flipo , B. Cortet , A. Tournadre , and J. Paccou , “Body Composition in Patients With Rheumatoid Arthritis: A Narrative Literature Review,” Therapeutic Advances in Musculoskeletal Disease 13 (2021): 1759720X211015006. 10.1177/1759720X211015006 PMC8221676 34221129

[114] G. A. Karpouzas , F. Cosedis Enevoldsen , P. Rezaeian , et al., “Pericoronary Adipose Tissue as a Biomarker of Coronary Atherosclerosis and Cardiovascular Risk in Rheumatoid Arthritis,” Rheumatology (Oxford, England) 65, no. 5 (2026): keag239. 42033781 10.1093/rheumatology/keag239 PMC13148959

[115] K. J. Shields , D. Stolz , S. C. Watkins , and J. M. Ahearn , “Complement Proteins C3 and C4 Bind to Collagen and Elastin in the Vascular Wall: A Potential Role in Vascular Stiffness and Atherosclerosis,” Clinical and Translational Science 4, no. 3 (2011): 146–152. 21707943 10.1111/j.1752-8062.2011.00304.x PMC5439861

[116] C. C. Ruan , D. L. Zhu , Q. Z. Chen , et al., “Perivascular Adipose Tissue‐Derived Complement 3 Is Required for Adventitial Fibroblast Functions and Adventitial Remodeling in Deoxycorticosterone Acetate‐Salt Hypertensive Rats,” Arteriosclerosis, Thrombosis, and Vascular Biology 30, no. 12 (2010): 2568–2574. 20864665 10.1161/ATVBAHA.110.215525

[117] K. Little , M. Llorian‐Salvador , M. Tang , et al., “Macrophage to Myofibroblast Transition Contributes to Subretinal Fibrosis Secondary to Neovascular Age‐Related Macular Degeneration,” Journal of Neuroinflammation 17, no. 1 (2020): 355. 33239022 10.1186/s12974-020-02033-7 PMC7690191

[118] R. Vittal , A. J. Fisher , E. L. Thompson , et al., “Overexpression of Decay Accelerating Factor Mitigates Fibrotic Responses to Lung Injury,” American Journal of Respiratory Cell and Molecular Biology 67, no. 4 (2022): 459–470. 35895592 10.1165/rcmb.2021-0463OC PMC9564933

[119] W. H. Yiu , R. X. Li , D. W. L. Wong , et al., “Complement C5a Inhibition Moderates Lipid Metabolism and Reduces Tubulointerstitial Fibrosis in Diabetic Nephropathy,” Nephrology, Dialysis, Transplantation 33, no. 8 (2018): 1323–1332. 10.1093/ndt/gfx336 29294056

[120] L. Janzic and K. Kouter , “Complement and Inflammasome Crosstalk in Chronic Inflammation,” Frontiers in Immunology 17 (2026): 1778759. 41983141 10.3389/fimmu.2026.1778759 PMC13070769

[121] E. Asgari , F. G. Le , H. Yamamoto , et al., “C3a Modulates IL‐1beta Secretion in Human Monocytes by Regulating ATP Efflux and Subsequent NLRP3 Inflammasome Activation,” Blood 122, no. 20 (2013): 3473–3481. 23878142 10.1182/blood-2013-05-502229

[122] E. O. Samstad , N. Niyonzima , S. Nymo , et al., “Cholesterol Crystals Induce Complement‐Dependent Inflammasome Activation and Cytokine Release,” Journal of Immunology 192, no. 6 (2014): 2837–2845. 10.4049/jimmunol.1302484 PMC3985066 24554772

[123] F. Laudisi , R. Spreafico , M. Evrard , et al., “Cutting Edge: The NLRP3 Inflammasome Links Complement‐Mediated Inflammation and IL‐1beta Release,” Journal of Immunology 191, no. 3 (2013): 1006–1010. 10.4049/jimmunol.1300489 PMC3718202 23817414

[124] C. D. Sadik , Y. Miyabe , T. Sezin , and A. D. Luster , “The Critical Role of C5a as an Initiator of Neutrophil‐Mediated Autoimmune Inflammation of the Joint and Skin,” Seminars in Immunology 37 (2018): 21–29. 29602515 10.1016/j.smim.2018.03.002

[125] Y. G. Tsai , Y. S. Wen , J. Y. Wang , et al., “Complement Regulatory Protein CD46 Induces Autophagy Against Oxidative Stress‐Mediated Apoptosis in Normal and Asthmatic Airway Epithelium,” Scientific Reports 8, no. 1 (2018): 12973. 30154478 10.1038/s41598-018-31317-5 PMC6113329

[126] I. Diaz‐Del‐Olmo , J. Worboys , F. Martin‐Sanchez , et al., “Internalization of the Membrane Attack Complex Triggers NLRP3 Inflammasome Activation and IL‐1beta Secretion in Human Macrophages,” Frontiers in Immunology 12 (2021): 720655. 34650553 10.3389/fimmu.2021.720655 PMC8506164

[127] X. Y. Wu , K. T. Li , H. X. Yang , et al., “Complement C1q Synergizes With PTX3 in Promoting NLRP3 Inflammasome Over‐Activation and Pyroptosis in Rheumatoid Arthritis,” Journal of Autoimmunity 106 (2020): 102336. 31601476 10.1016/j.jaut.2019.102336

[128] L. Wen , D. Bi , and Y. Shen , “Complement‐Mediated Synapse Loss in Alzheimer's Disease: Mechanisms and Involvement of Risk Factors,” Trends in Neurosciences 47, no. 2 (2024): 135–149. 38129195 10.1016/j.tins.2023.11.010

[129] V. S. Ten , S. A. Sosunov , S. P. Mazer , et al., “C1q‐Deficiency Is Neuroprotective Against Hypoxic‐Ischemic Brain Injury in Neonatal Mice,” Stroke 36, no. 10 (2005): 2244–2250. 16179576 10.1161/01.STR.0000182237.20807.d0

[130] X. Zhao , C. Wang , B. Pang , Y. Zhu , and Y. Zhang , “The Value of Serum Complement C1q in the Diagnosis of Acute Ischemic Stroke,” Clinical Laboratory 63, no. 5 (2017): 915–920. 28627821 10.7754/Clin.Lab.2016.161033

[131] M. E. Benoit , E. V. Clarke , P. Morgado , D. A. Fraser , and A. J. Tenner , “Complement Protein C1q Directs Macrophage Polarization and Limits Inflammasome Activity During the Uptake of Apoptotic Cells,” Journal of Immunology 188, no. 11 (2012): 5682–5693. 10.4049/jimmunol.1103760 PMC3358549 22523386

[132] M. C. Pulanco , J. Cosman , M. M. Ho , et al., “Complement Protein C1q Enhances Macrophage Foam Cell Survival and Efferocytosis,” Journal of Immunology 198, no. 1 (2017): 472–480. 10.4049/jimmunol.1601445 PMC5173410 27895181

[133] G. Cavaliere , F. Cimmino , G. Trinchese , et al., “From Obesity‐Induced Low‐Grade Inflammation to Lipotoxicity and Mitochondrial Dysfunction: Altered Multi‐Crosstalk Between Adipose Tissue and Metabolically Active Organs,” Antioxidants 12, no. 6 (2023): 1172. 37371902 10.3390/antiox12061172 PMC10295335

[134] C. Drummer , F. Saaoud , N. C. Jhala , et al., “Caspase‐11 Promotes High‐Fat Diet‐Induced NAFLD by Increasing Glycolysis, OXPHOS, and Pyroptosis in Macrophages,” Frontiers in Immunology 14 (2023): 1113883. 36776889 10.3389/fimmu.2023.1113883 PMC9909353

[135] G. Arbore and C. Kemper , “A Novel “Complement‐Metabolism‐Inflammasome Axis” as a Key Regulator of Immune Cell Effector Function,” European Journal of Immunology 46, no. 7 (2016): 1563–1573. 27184294 10.1002/eji.201546131 PMC5025719

[136] G. Arbore , E. E. West , R. Spolski , et al., “T Helper 1 Immunity Requires Complement‐Driven NLRP3 Inflammasome Activity in CD4(+) T Cells,” Science 352, no. 6292 (2016): aad1210. 27313051 10.1126/science.aad1210 PMC5015487

[137] E. E. West and C. Kemper , “Complosome‐the Intracellular Complement System,” Nature Reviews. Nephrology 19, no. 7 (2023): 426–439. 37055581 10.1038/s41581-023-00704-1 PMC10100629

[138] T. Teissier and E. Boulanger , “The Receptor for Advanced Glycation End‐Products (RAGE) is an Important Pattern Recognition Receptor (PRR) for Inflammaging,” Biogerontology 20, no. 3 (2019): 279–301. 30968282 10.1007/s10522-019-09808-3

[139] Y. Mamane , C. Chung Chan , G. Lavallee , et al., “The C3a Anaphylatoxin Receptor is a Key Mediator of Insulin Resistance and Functions by Modulating Adipose Tissue Macrophage Infiltration and Activation,” Diabetes 58, no. 9 (2009): 2006–2017. 19581423 10.2337/db09-0323 PMC2731537

[140] J. Phieler , K. J. Chung , A. Chatzigeorgiou , et al., “The Complement Anaphylatoxin C5a Receptor Contributes to Obese Adipose Tissue Inflammation and Insulin Resistance,” Journal of Immunology 191, no. 8 (2013): 4367–4374. 10.4049/jimmunol.1300038 PMC3817864 24043887

[141] A. Muscari , C. Bozzoli , G. M. Puddu , et al., “Association of Serum C3 Levels With the Risk of Myocardial Infarction,” American Journal of Medicine 98, no. 4 (1995): 357–364. 7709948 10.1016/S0002-9343(99)80314-3

[142] G. Engstrom , B. Hedblad , L. Janzon , and F. Lindgarde , “Weight Gain in Relation to Plasma Levels of Complement Factor 3: Results From a Population‐Based Cohort Study,” Diabetologia 48, no. 12 (2005): 2525–2531. 16283247 10.1007/s00125-005-0021-6

[143] Y. Xin , E. Hertle , C. J. H. van der Kallen , C. G. Schalkwijk , C. D. A. Stehouwer , and M. M. J. van Greevenbroek , “Complement C3 and C4, but Not Their Regulators or Activated Products, Are Associated With Incident Metabolic Syndrome: The CODAM Study,” Endocrine 62, no. 3 (2018): 617–627. 30132263 10.1007/s12020-018-1712-3 PMC6244913

[144] N. Wlazlo , M. M. van Greevenbroek , I. Ferreira , et al., “Complement Factor 3 Is Associated With Insulin Resistance and With Incident Type 2 Diabetes Over a 7‐Year Follow‐Up Period: The CODAM Study,” Diabetes Care 37, no. 7 (2014): 1900–1909. 24760264 10.2337/dc13-2804

[145] M. Botto , C. Dell'Agnola , A. E. Bygrave , et al., “Homozygous C1q Deficiency Causes Glomerulonephritis Associated With Multiple Apoptotic Bodies,” Nature Genetics 19, no. 1 (1998): 56–59. 9590289 10.1038/ng0598-56

[146] C. Yin , S. Ackermann , Z. Ma , et al., “ApoE Attenuates Unresolvable Inflammation by Complex Formation With Activated C1q,” Nature Medicine 25, no. 3 (2019): 496–506. 10.1038/s41591-018-0336-8 PMC6420126 30692699

[147] W. Ma , V. Rai , B. I. Hudson , F. Song , A. M. Schmidt , and G. R. Barile , “RAGE Binds C1q and Enhances C1q‐Mediated Phagocytosis,” Cellular Immunology 274, no. 1–2 (2012): 72–82. 22386596 10.1016/j.cellimm.2012.02.001

[148] U. Andersson , H. Yang , and H. Harris , “High‐Mobility Group Box 1 Protein (HMGB1) Operates as an Alarmin Outside as Well as Inside Cells,” Seminars in Immunology 38 (2018): 40–48. 29530410 10.1016/j.smim.2018.02.011

[149] M. Son , A. Porat , M. He , et al., “C1q and HMGB1 Reciprocally Regulate Human Macrophage Polarization,” Blood 128, no. 18 (2016): 2218–2228. 27683415 10.1182/blood-2016-05-719757 PMC5095756

[150] T. Liu , A. Xiang , T. Peng , et al., “HMGB1‐C1q Complexes Regulate Macrophage Function by Switching Between Leukotriene and Specialized Proresolving Mediator Biosynthesis,” Proceedings of the National Academy of Sciences of the United States of America 116, no. 46 (2019): 23254–23263. 31570601 10.1073/pnas.1907490116 PMC6859319

[151] E. Hertle , I. C. W. Arts , C. J. H. van der Kallen , et al., “Classical Pathway of Complement Activation: Longitudinal Associations of C1q and C1‐INH With Cardiovascular Outcomes: The CODAM Study (Cohort on Diabetes and Atherosclerosis Maastricht)‐Brief Report,” Arteriosclerosis, Thrombosis, and Vascular Biology 38, no. 5 (2018): 1242–1244. 29567681 10.1161/ATVBAHA.118.310806

[152] L. N. Troelsen , P. Garred , B. Christiansen , et al., “Double Role of Mannose‐Binding Lectin in Relation to Carotid Intima‐Media Thickness in Patients With Rheumatoid Arthritis,” Molecular Immunology 47, no. 4 (2010): 713–718. 19939454 10.1016/j.molimm.2009.10.021

[153] C. Prugger , G. Luc , B. Haas , et al., “Adipocytokines and the Risk of Ischemic Stroke: The PRIME STUDY,” Annals of Neurology 71, no. 4 (2012): 478–486. 22522440 10.1002/ana.22669

[154] G. Luc , J. P. Empana , P. Morange , et al., “Adipocytokines and the Risk of Coronary Heart Disease in Healthy Middle Aged Men: The PRIME Study,” International Journal of Obesity 34, no. 1 (2010): 118–126. 19823188 10.1038/ijo.2009.204

[155] S. Jin , S. Eussen , C. G. Schalkwijk , C. D. A. Stehouwer , and M. M. J. van Greevenbroek , “Plasma Factor D is Cross‐Sectionally Associated With Low‐Grade Inflammation, Endothelial Dysfunction and Cardiovascular Disease: The Maastricht Study,” Atherosclerosis 377 (2023): 60–67. 37406499 10.1016/j.atherosclerosis.2023.06.079

[156] N. D. Schartz , H. Y. Liang , K. Carvalho , et al., “C5aR1 Antagonism Suppresses Inflammatory Glial Responses and Alters Cellular Signaling in an Alzheimer's Disease Mouse Model,” Nature Communications 15, no. 1 (2024): 7028. 10.1038/s41467-024-51163-6 PMC11327341 39147742

[157] L. L. Wei , N. Ma , K. Y. Wu , et al., “Protective Role of C3aR (C3a Anaphylatoxin Receptor) Against Atherosclerosis in Atherosclerosis‐Prone Mice,” Arteriosclerosis, Thrombosis, and Vascular Biology 40, no. 9 (2020): 2070–2083. 32762445 10.1161/ATVBAHA.120.314150

[158] N. Niyonzima , J. Rahman , N. Kunz , et al., “Mitochondrial C5aR1 Activity in Macrophages Controls IL‐1β Production Underlying Sterile Inflammation,” Science Immunology 6, no. 66 (2021): eabf2489. 34932384 10.1126/sciimmunol.abf2489 PMC8902698

[159] K. Pilely , A. Rosbjerg , N. Genster , et al., “Cholesterol Crystals Activate the Lectin Complement Pathway via Ficolin‐2 and Mannose‐Binding Lectin: Implications for the Progression of Atherosclerosis,” Journal of Immunology 196, no. 12 (2016): 5064–5074. 10.4049/jimmunol.1502595 27183610

[160] E. Vataja , G. Ratti , A. Safa , et al., “Exploring the Intersection of Atherosclerosis and Alzheimer's Disease: The Role of Inflammation and Complement Activation,” Inflammation Research 74, no. 1 (2025): 102. 40637922 10.1007/s00011-025-02069-6 PMC12245954

[161] M. G. Kiss and C. J. Binder , “The Multifaceted Impact of Complement on Atherosclerosis,” Atherosclerosis 351 (2022): 29–40. 35365353 10.1016/j.atherosclerosis.2022.03.014

[162] S. O'Barr and N. R. Cooper , “The C5a Complement Activation Peptide Increases IL‐1beta and IL‐6 Release From Amyloid‐Beta Primed Human Monocytes: Implications for Alzheimer's Disease,” Journal of Neuroimmunology 109, no. 2 (2000): 87–94. 10996210 10.1016/s0165-5728(00)00291-5

[163] K. Kulak , G. T. Westermark , N. Papac‐Milicevic , E. Renström , A. M. Blom , and B. C. King , “The Human Serum Protein C4b‐Binding Protein Inhibits Pancreatic IAPP‐Induced Inflammasome Activation,” Diabetologia 60, no. 8 (2017): 1522–1533. 28500395 10.1007/s00125-017-4286-3 PMC5491568

[164] T. Q. Ge , P. P. Guan , and P. Wang , “Complement 3a Induces the Synapse Loss via C3aR in Mitochondria‐Dependent NLRP3 Activating Mechanisms During the Development and Progression of Alzheimer's Disease,” Neuroscience and Biobehavioral Reviews 165 (2024): 105868. 39218048 10.1016/j.neubiorev.2024.105868

[165] N. E. Propson , E. R. Roy , A. Litvinchuk , J. Köhl , and H. Zheng , “Endothelial C3a Receptor Mediates Vascular Inflammation and Blood‐Brain Barrier Permeability During Aging,” Journal of Clinical Investigation 131, no. 1 (2021): e140966. 32990682 10.1172/JCI140966 PMC7773352

[166] S. H. Krance , C. Y. Wu , Y. Zou , et al., “The Complement Cascade in Alzheimer's Disease: A Systematic Review and Meta‐Analysis,” Molecular Psychiatry 26, no. 10 (2021): 5532–5541. 31628417 10.1038/s41380-019-0536-8

[167] M. Hernández‐Díaz , D. Rodríguez‐González , E. Heras‐Recuero , et al., “The Relationship Between the Complement System and Subclinical Carotid Atherosclerosis in Patients With Rheumatoid Arthritis,” Arthritis Research & Therapy 26, no. 1 (2024): 127. 38978073 10.1186/s13075-024-03360-3 PMC11229295

[168] K. L. Rasmussen , B. G. Nordestgaard , R. Frikke‐Schmidt , and S. F. Nielsen , “An Updated Alzheimer Hypothesis: Complement C3 and Risk of Alzheimer's Disease‐A Cohort Study of 95,442 Individuals,” Alzheimer's & Dementia 14, no. 12 (2018): 1589–1601. 10.1016/j.jalz.2018.07.223 30243924

[169] M. Maier , Y. Peng , L. Jiang , T. J. Seabrook , M. C. Carroll , and C. A. Lemere , “Complement C3 Deficiency Leads to Accelerated Amyloid Beta Plaque Deposition and Neurodegeneration and Modulation of the Microglia/Macrophage Phenotype in Amyloid Precursor Protein Transgenic Mice,” Journal of Neuroscience 28, no. 25 (2008): 6333–6341. 18562603 10.1523/JNEUROSCI.0829-08.2008 PMC3329761

[170] L. Persson , J. Borén , A. K. Robertson , V. Wallenius , G. K. Hansson , and M. Pekna , “Lack of Complement Factor C3, but Not Factor B, Increases Hyperlipidemia and Atherosclerosis in Apolipoprotein E−/− Low‐Density Lipoprotein Receptor−/− Mice,” Arteriosclerosis, Thrombosis, and Vascular Biology 24, no. 6 (2004): 1062–1067. 15059809 10.1161/01.ATV.0000127302.24266.40

[171] P. Triggianese and D. Della‐Morte , “The Inner Fire: The Shifting Paradigm of Complement System in Aging,” Frontiers in Immunology 17 (2026): 1795833. 42058191 10.3389/fimmu.2026.1795833 PMC13121120

[172] D. A. Towler , C. M. Giachelli , M. Scatena , Y. Tintut , and L. L. Demer , “Calcific Aortopathy in Response to Aging and Injury,” Circulation 153, no. 10 (2026): 769–785. 41802024 10.1161/CIRCULATIONAHA.125.072393 PMC12965821

[173] S. Nagy , J. Ditchek , and M. M. Kesselman , “Coronary Artery Calcification in Rheumatoid Arthritis Patients: A Systematic Review,” Cureus 16, no. 9 (2024): e70517. 39479072 10.7759/cureus.70517 PMC11524640

[174] M. L. Gross , H. P. Meyer , H. Ziebart , et al., “Calcification of Coronary Intima and Media: Immunohistochemistry, Backscatter Imaging, and X‐Ray Analysis in Renal and Nonrenal Patients,” Clinical Journal of the American Society of Nephrology 2, no. 1 (2007): 121–134. 17699396 10.2215/CJN.01760506

[175] J. Lim , J. T. Aguilan , R. S. Sellers , et al., “Lipid Mass Spectrometry Imaging And Proteomic Analysis of Severe Aortic Stenosis,” Journal of Molecular Histology 51, no. 5 (2020): 559–571. 32794037 10.1007/s10735-020-09905-5 PMC7672660

[176] K. Manger , M. Kusus , C. Forster , et al., “Factors Associated With Coronary Artery Calcification in Young Female Patients With SLE,” Annals of the Rheumatic Diseases 62, no. 9 (2003): 846–850. 12922957 10.1136/ard.62.9.846 PMC1754656

[177] Y. Wang , Y. Miao , K. Gong , X. Cheng , Y. Chen , and M. H. Zhao , “Plasma Complement Protein C3a Level Was Associated With Abdominal Aortic Calcification in Patients on Hemodialysis,” Journal of Cardiovascular Translational Research 12, no. 5 (2019): 496–505. 30989586 10.1007/s12265-019-09885-2

[178] A. Liu , P. Luo , and H. Huang , “New Insight of Complement System in the Process of Vascular Calcification,” Journal of Cellular and Molecular Medicine 27, no. 9 (2023): 1168–1178. 37002701 10.1111/jcmm.17732 PMC10148053

[179] P. Carrera‐Bastos , A. Plaza‐Florido , A. Santos‐Lozano , et al., “The ‘Autoimmunome’ of Centenarians,” J Transl Autoimmun 11 (2025): 100295. 40546301 10.1016/j.jtauto.2025.100295 PMC12182348

[180] S. Watanabe , K. Sato , N. Hasegawa , et al., “Serum C1q as a Novel Biomarker of Sarcopenia in Older Adults,” FASEB Journal 29, no. 3 (2015): 1003–1010. 25491308 10.1096/fj.14-262154

[181] E. C. Pardali , M. Klonizakis , D. G. Goulis , et al., “Sarcopenia in Rheumatic Diseases: A Hidden Issue of Concern,” Diseases 13, no. 5 (2025): 134. 40422566 10.3390/diseases13050134 PMC12110307

[182] R. J. Delfino , N. Staimer , and N. D. Vaziri , “Air Pollution and Circulating Biomarkers of Oxidative Stress,” Air Quality, Atmosphere and Health 4, no. 1 (2011): 37–52. 10.1007/s11869-010-0095-2 PMC3634798 23626660

[183] Z. Kang , H. Li , G. Li , and D. Yin , “Reaction of Pyridoxamine With Malondialdehyde: Mechanism of Inhibition of Formation of Advanced Lipoxidation End‐Products,” Amino Acids 30, no. 1 (2006): 55–61. 15990947 10.1007/s00726-005-0209-6

[184] M. D. G. Van den Eynde , J. M. Geleijnse , J. Scheijen , et al., “Quercetin, but Not Epicatechin, Decreases Plasma Concentrations of Methylglyoxal in Adults in a Randomized, Double‐Blind, Placebo‐Controlled, Crossover Trial With Pure Flavonoids,” Journal of Nutrition 148, no. 12 (2018): 1911–1916. 30398646 10.1093/jn/nxy236

[185] L. Ying , M. T. Chaudhry , F. Xiao , et al., “The Effects and Mechanism of Quercetin Dietary Supplementation in Streptozotocin‐Induced Hyperglycemic Arbor Acre Broilers,” Oxidative Medicine and Cellular Longevity 2020 (2020): 9585047. 32104545 10.1155/2020/9585047 PMC7035566

[186] S. Ilari , S. Proietti , F. Milani , et al., “Dietary Patterns, Oxidative Stress, and Early Inflammation: A Systematic Review and Meta‐Analysis Comparing Mediterranean, Vegan, and Vegetarian Diets,” Nutrients 17, no. 3 (2025): 548. 39940408 10.3390/nu17030548 PMC11819869

[187] R. Militello , S. Luti , T. Gamberi , A. Pellegrino , A. Modesti , and P. A. Modesti , “Physical Activity and Oxidative Stress in Aging,” Antioxidants (Basel) 13, no. 5 (2024): 557. 38790662 10.3390/antiox13050557 PMC11117672

[188] R. Talhout , A. Opperhuizen , and J. G. van Amsterdam , “Role of Acetaldehyde in Tobacco Smoke Addiction,” European Neuropsychopharmacology 17, no. 10 (2007): 627–636. 17382522 10.1016/j.euroneuro.2007.02.013

[189] J. Scheijen , E. Clevers , L. Engelen , et al., “Analysis of Advanced Glycation Endproducts in Selected Food Items by Ultra‐Performance Liquid Chromatography Tandem Mass Spectrometry: Presentation of a Dietary AGE Database,” Food Chemistry 190 (2016): 1145–1150. 26213088 10.1016/j.foodchem.2015.06.049

[190] X. Lu , R. Ma , J. Zhan , et al., “Effect of Dietary Intake of Advanced Glycation End Products on Biomarkers of Type 2 Diabetes: A Systematic Review and Meta‐Analysis,” Critical Reviews in Food Science and Nutrition 65, no. 26 (2025): 5277–5286. 39320860 10.1080/10408398.2024.2407894

[191] K. Maasen , A. L. Mayen , C. Hana , et al., “Higher Intake of Dietary Dicarbonyl Compounds Is Associated With Lower Incidence of Type 2 Diabetes: European Prospective Investigation Into Cancer and Nutrition (EPIC)‐InterAct Case‐Cohort Study,” European Journal of Nutrition 65, no. 3 (2026): 98. 41843191 10.1007/s00394-026-03904-0 PMC12996025

[192] A. L. Mayén , K. Maasen , C. Hana , et al., “Higher Intakes of Dietary Dicarbonyl Compounds Are Associated With Lower Risk of Cardiovascular Disease,” European Journal of Preventive Cardiology 32, no. 16 (2025): 1588–1600. 40036164 10.1093/eurjpc/zwaf060 PMC12600303

[193] WHO , https://www.who.int/europe/news‐room/fact‐sheets/item/everyday‐actions‐for‐better‐health‐who‐recommendations.

[194] D. M. Boeters , L. E. Burgers , R. E. Toes , and A. van der Helm‐van Mil , “Does Immunological Remission, Defined as Disappearance of Autoantibodies, Occur With Current Treatment Strategies? A Long‐Term Follow‐Up Study in Rheumatoid Arthritis Patients Who Achieved Sustained DMARD‐Free Status,” Annals of the Rheumatic Diseases 78, no. 11 (2019): 1497–1504. 31413004 10.1136/annrheumdis-2018-214868 PMC7212006

[195] A. Vancsa , Z. Szabo , S. Szamosi , et al., “Longterm Effects of Rituximab on B Cell Counts and Autoantibody Production in Rheumatoid Arthritis: Use of High‐Sensitivity Flow Cytometry for More Sensitive Assessment of B Cell Depletion,” Journal of Rheumatology 40, no. 5 (2013): 565–571. 23547216 10.3899/jrheum.111488

[196] D. Mougiakakos , G. Kronke , S. Volkl , et al., “CD19‐Targeted CAR T Cells in Refractory Systemic Lupus Erythematosus,” New England Journal of Medicine 385, no. 6 (2021): 567–569. 34347960 10.1056/NEJMc2107725

[197] P. Kremer , I. Haase , J. Richter , et al., “A New Therapeutic Frontier: CD19‐Targeting CAR‐T Cell Therapy in Rheumatoid Arthritis: A Concise Report,” Rheumatology (Oxford, England) 65 (2026): keag195. 41984818 10.1093/rheumatology/keag195

[198] M. Lidar , D. Rimar , P. David , et al., “CD‐19 CAR‐T Cells for Polyrefractory Rheumatoid Arthritis,” Annals of the Rheumatic Diseases 84, no. 2 (2025): 370–372. 39919909 10.1136/ard-2024-226437

[199] M. Mannes , W. M. Zelek , and L. A. Trouw , “Shifting From Systemic to Precision‐Targeted Complement Therapies: Opportunities and Hurdles,” European Journal of Immunology 56, no. 6 (2026): e70231, 10.1002/eji.70231. 42319133 PMC13281374

[200] L. A. Trouw , M. C. Pickering , and A. M. Blom , “The Complement System as a Potential Therapeutic Target in Rheumatic Disease,” Nature Reviews Rheumatology 13, no. 9 (2017): 538–547. 28794515 10.1038/nrrheum.2017.125

[201] F. Liu , S. Wawersik , S. Tomlinson , J. M. Thurman , and V. M. Holers , “Tissue‐Targeted Regulators of Complement for Amelioration of Human Disease: Rationale and Novel Therapeutic Strategies,” Journal of Immunology 214, no. 9 (2025): 2138–2149. 10.1093/jimmun/vkaf053 40258303

[202] H. Wang , D. J. van den Brink , B. A. Heesters , P. W. Parren , and L. A. Trouw , “Local Complement Inhibition by Selective Precision‐Targeted Therapies,” Current Opinion in Immunology 98 (2026): 102687. 41166772 10.1016/j.coi.2025.102687

[203] F. Liu , S. T. Ryan , K. C. Fahnoe , et al., “C3d‐Targeted Factor H Inhibits Tissue Complement in Disease Models and Reduces Glomerular Injury Without Affecting Circulating Complement,” Molecular Therapy 32, no. 4 (2024): 1061–1079. 38382529 10.1016/j.ymthe.2024.02.001 PMC11163200

[204] S. Tomlinson and J. M. Thurman , “Tissue‐Targeted Complement Therapeutics,” Molecular Immunology 102 (2018): 120–128. 30220307 10.1016/j.molimm.2018.06.005 PMC6146401

[205] H. Wang , F. S. van de Bovenkamp , D. J. Dijkstra , et al., “Targeted Complement Inhibition Using Bispecific Antibodies That Bind Local Antigens and Endogenous Complement Regulators,” Frontiers in Immunology 15 (2024): 1288597. 38817607 10.3389/fimmu.2024.1288597 PMC11137741

